# Structural Insight of New Butyrylcholinesterase Inhibitors Based on Benzylbenzofuran Scaffold

**DOI:** 10.3390/ph15030304

**Published:** 2022-03-02

**Authors:** Giovanna L. Delogu, Antonella Fais, Francesca Pintus, Chinmayi Goyal, Maria J. Matos, Benedetta Era, Amit Kumar

**Affiliations:** 1 Department of Life and Environmental Sciences, University of Cagliari, Monserrato, 09042 Cagliari, Italy; delogug@unica.it (G.L.D.); fais@unica.it (A.F.); fpintus@unica.it (F.P.); 2 Yorktown High School, 2727 Crompond Road, Yorktown Heights, NY 10598, USA; chinmayigoyal.1@gmail.com; 3 Departamento de Quìmica Orgànica, Facultade de Farmacia, Universidade de Santiago de Compostela, 15782 Santiago de Compostela, Spain; mariacmatos@gmail.com; 4 Department of Electrical and Electronic Engineering, University of Cagliari, Via Marengo 2, 09123 Cagliari, Italy; amit369@gmail.com

**Keywords:** benzylbenzofuran, butyrylcholinesterase inhibitors, docking studies

## Abstract

In the present work, we use a merger of computational and biochemical techniques as a rational guideline for structural modification of benzofuran derivatives to find pertinent structural features for the butyrylcholinesterase inhibitory activity and selectivity. Previously, we revealed a series of 2-phenylbenzofuran compounds that displayed a selective inhibitory activity for BChE. Here, in an effort to discover novel selective BChE inhibitors with favorable physicochemical and pharmacokinetic profiles, 2-benzylbenzofurans were designed, synthesized, and evaluated as BChE inhibitors. The 2-phenylbenzofuran scaffold structure is modified by introducing one methylene spacer between the benzofuran core and the 2-phenyl ring with a hydroxyl substituent in the para or meta position. Either position 5 or 7 of the benzofuran scaffold was substituted with a bromine or chlorine atom. Further assessment of the selected list of compounds indicated that the substituent’s nature and position determined their activity and selectivity. 5-bromo-2-(4-hydroxybenzyl)benzofuran **9B** proved to be the most potent butyrylcholinesterase inhibitor (IC_50_ = 2.93 µM) of the studied series. Computational studies were carried out to correlate the theoretical and experimental binding affinity of the compounds to the BChE protein.

## 1. Introduction

The design of enzyme inhibitors has been a topic of accelerated research, especially concerning the discovery of lead compounds as new therapeutic agents owing to the weak pharmacokinetic properties of several bioactive molecules.

Molecular docking is a popular tool in computer-aided drug discovery for rapid screening and predicting the binding characteristics of small molecules to a target protein using its available three-dimensional structure. The action as inhibitors of several molecules is related to their target enzyme interaction. The rational drug design strategy is supported by a detailed understanding of interactions between small molecules and proteins [1,2]. 

New molecules are conceived either based on similarities with known reference structures or their complementarity with the 3D structure of known active sites.

The beginning for the rational design of inhibitors of enzymes is existing drugs and known pharmacophores. In this regard, benzofuran scaffolds have drawn considerable attention over the last few years due to their deep biological properties, as well as their widespread occurrence in nature. 

Many works have delineated that 2-arylbenzofuran compounds have tenacious biological activities—such as antibacterial [3], anti-oxidative [4], anti-tumour [5], anti-viral, and anti-inflammatory properties [6]. The numerous biological latent features in the benzofuran scaffold justify the wide attraction in accepting benzofuran as building blocks of pharmacological medicine. Many of the clinically approved drugs are synthetic or naturally occurring substituted benzofuran derivatives, some of which have other heterocyclic fractions in structure [7]. 

In our recent studies, we reported that 2-phenylbenzofuran derivatives display promising enzymatic inhibition properties against BChE [8,9,10] and monoamine oxidase (MAO) [11,12,13] enzymes. BChE plays a key role in the progression of Alzheimer’s disease (AD), a neurodegenerative disorder. Among many factors that are involved in AD pathogenesis, the primary ones comprise alterations of the cholinergic system; hyperproduction and aggregation of amyloid beta neurotoxic peptide (Aβ); and oxidative stress and aggregation of the Aβ peptide [14,15]. The AChE and BChE inhibition have been documented as a target for the effective management of AD by an increase in the availability of acetylcholine in the brain regions and a decrease in the Aβ deposition [16]. In recent years, there has been ongoing research into the synthesis of more potent and highly effective cholinesterase inhibitors [17]. 

Our data showed that 2-phenylbenzofuran derivatives with a hydroxyl group at the meta position in the phenyl ring and substitution with bromine and chlorine at position 7 in the benzofuran ring resulted in a high inhibition value. Notably, molecular docking revealed that these compounds interact with two major substrate-binding sites of BChE. The benzofuran moiety interacted with peripheral anionic site (PAS) residues and the 2-phenyl ring moiety with catalytic anionic site (CAS) residues [10].

Recently, the authors reported a new series of 2-benzylbenzofuran isolated from natural sources that showed inhibitory activity for acetylcholinesterase [18]. Hence, we decided to introduce one methylene spacer between the benzofuran core and the 2-phenyl ring with the aim of improving the flexibility of compounds and positively influencing their inhibitory activity against the BChE enzyme. 

## 2. Results and Discussion

### 2.1. Chemistry

**Scheme 1:**
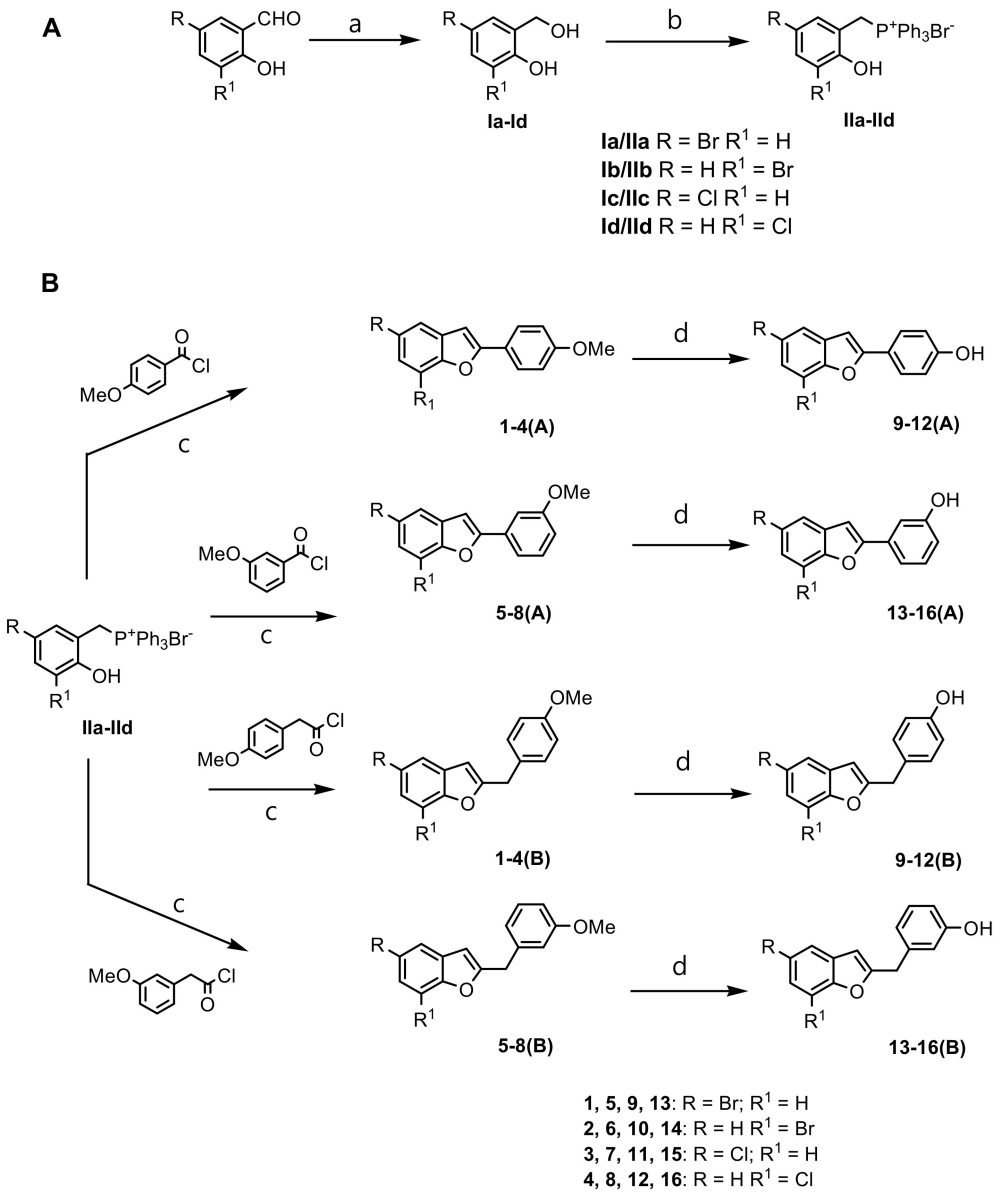
Synthesis via Wittig reaction of 2-phenylbenzofuran derivatives **1**–**16**(**A**) and 2-benzylbenzofuran derivatives **1**–**16**(**B**). Reagents and conditions: (**A**) (a) NaBH_4_, EtOH, 0 °C to rt, 2 h; (b) PPh_3_ HBr, CH_3_CN, 82 °C, 2 h; (**B**) (c) toluene, Et_3_N, 110 °C, 2 h; (d) HI/AcOH/Ac_2_O, 0 °C to reflux, 4 h.

2-Phenylbenzofurans **1**–**8**(**A**) and 2-benzylbenzofurans **1**–**8**(**B**) derivatives were produced efficiently by applying an intramolecular Wittig reaction (Scheme 1). 

Ortho-hydroxybenzyl alcohols **Ia**–**Id** [19] were taken as the starting material. The first step involved the formation of Wittig reagents 2-hydroxybenzyltriphosphonium salts **IIa**–**IId** from **Ia**–**Id**.

The benzofuran ring formation was reached by a reaction between phosphonium salts **IIa**–**IId** and convenient acyl chlorides [8,9,10].

Hydrolysis of the methoxy group of compounds **1**–**8**(**A**) and **1**–**8**(**B**) was done by treatment with hydrogen iodide in acetic acid/acetic anhydride to provide, in almost quantitative yields, the corresponding hydroxy derivatives **9**–**16**(**A**) and **9**–**16**(**B**) [20]. The benzofuran structures were approved by spectroscopic techniques such as ^1^H NMR, ^13^C NMR, and elemental analysis (Supporting Information—1. Chemistry).

### 2.2. Biological Activity

The IC_50_ values of benzofuran derivatives for BChE inhibition are illustrated in Table 1. The effect of the benzofuran derivatives was evaluated on the commercial enzyme from an animal source due to its low cost and remarkable similarity with the human enzyme. 

Among derivatives of para-hydroxy substituted **9**–**12**(**B**), the most active compound is 5-bromo-2-(4-hydroxybenzyl) benzofuran (**9B**) with an IC_50_ value of 2.93 µM and with an inhibitory activity 10 times higher than galantamine (IC_50_ = 28.06 µM), the reference inhibitor. Benzylbenzofuran isomer **10B**, with the bromine atom in position 7 in the benzofuran ring, is less active than the previous one, but it has similar inhibitory activity to galantamine. Compounds **11B** and **12B,** with the chlorine atom in positions 5 and 7 respectively, are the least active of this series. In the series of 2-benzylbenzofurans meta-hydroxy substituted **13**–**16**(**B**) the most active compound is 5-chloro-2-(3-hydroxybenzyl)benzofuran (**15B**, IC_50_ = 27.84 µM).

In Table 1, these data are compared with IC_50_ values of the synthesized 2-phenylbenzofurans **9**–**16**(**A**) [8,10]. Overall, the 2-benzylbenzofuran derivatives substituted in para-position **9**–**11**(**B**) showed better inhibitory activity against BChE than 2-phenylbenzofurans **9**–**11**(**A**). Only compound **12B** displayed a lower inhibitory activity than compound **12A**. All 2-benzylbenzofuran derivatives substituted in meta-position **13**–**16**(**B**) show lower inhibitory activity than 2-phenylbenzofurans **13**–**16**(**A**).

5-bromo-2-(4-hydroxybenzyl)benzofuran (**9B**) exhibited about 4-fold higher inhibitory activity than 7-bromo-2-(3-hydroxyphenyl)benzofuran (**14A**), which showed the greatest enzyme inhibitory activity among the 2-phenylbenzofuran derivatives.

Compound **9B** has a statistically different IC_50_ value from the other compounds, making it the most active of the whole series. For the active compound (**9B**), the presence of the methylene spacer between the benzofuran core and the 2-phenyl ring was revealed to be fundamental for inhibitory activity. This modification probably allows the molecule to adapt better in the enzymatic pocket and form a more significant interaction with the protein.

This hypothesis could be confirmed by comparing IC_50_ value of the 2-benzylbenzofuran **9B** (compound with the highest activity) and the 2-phenylbenzofuran **9A** (with no activity), which differ only in the methylene spacer.

### 2.3. Molecular and Physicochemical Properties of Compounds

Molecular docking is a proven computational method to identify the most probable orientation between a small molecule (compound) and a protein, human hBChE. In this study, we aimed to enhance the flexibility of compounds to improve inhibitory activity against the BChE enzyme.

Specifically, compounds **9A** and **10A**—which showed negligible inhibitory activity—were modified respectively to compounds **9B** and **10B**. Experimental results suggested an improvement in enzyme inhibition for compounds **9B** and **10B**. Therefore, molecular docking was performed for compounds (**9A**, **9B**, **10A**, and **10B**) to understand better the outcome of adding a methylene spacer on the compound’s activity against hBChE protein. Docking results (Table 2) suggested superior docking energy values for the compounds with a methylene spacer, and among them, compound **9B** displayed the best docking energy value against the target protein hBChE.

**Figure 1:**
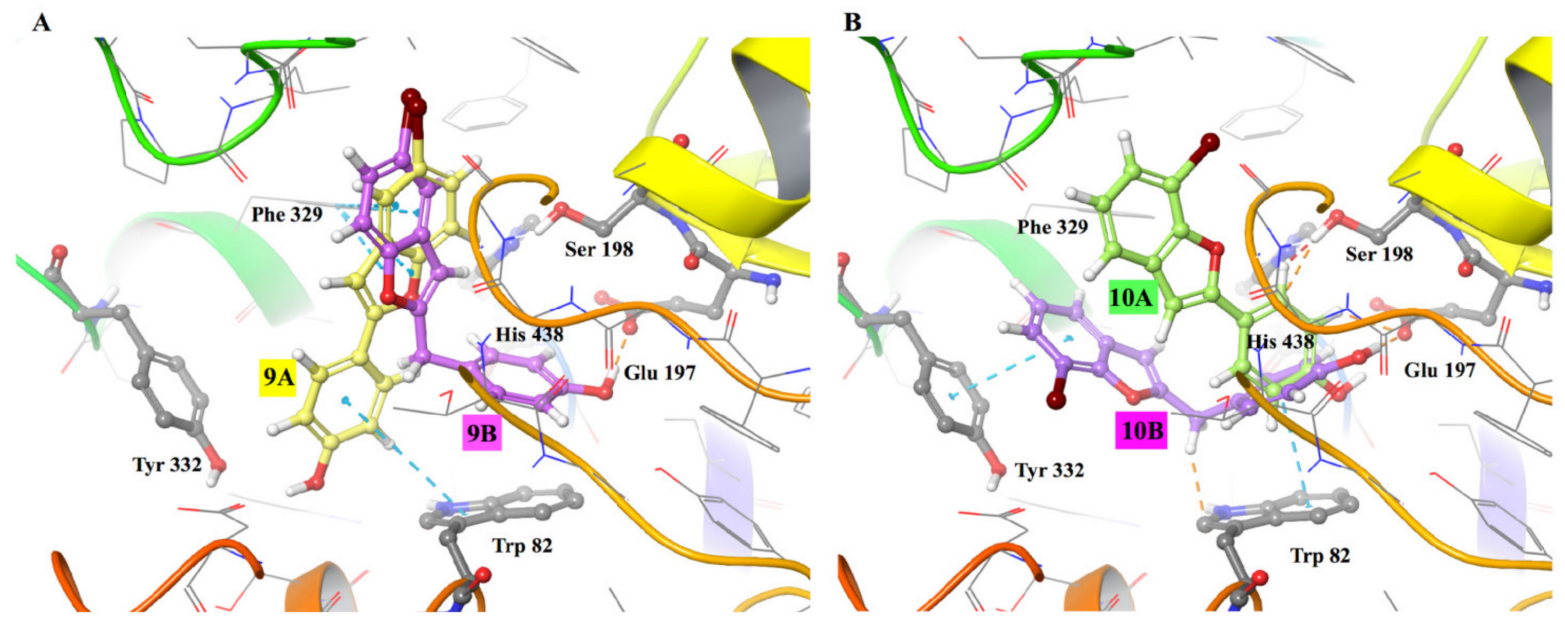
Protein-ligand interaction picture. (**A**) The binding site of compounds **9A** and **9B**; and in (**B**) for compounds **10A** and **10B**. The key interacting residues are shown in ball-and-stick representation.

The docking results of the investigated compound are in good agreement with the experimental IC_50_ values. In Figure 1, we show the interaction picture for the investigated protein–ligand complexes.

We note an adequate overlap of the benzofuran moiety of compounds **9A** and **9B** (Figure 1A) and pi–pi interactions with Phe 329. The presence of methylene spacer in compound **9B** alters the orientation of the 2-phenyl group compared to compound **9A**, such that it can readily nestle inside the catalytic subsite and non-covalently interact with residues Glu 197, Ser 198, and His 438 (Figure 1A). Thus, we can deduce that introducing a methylene spacer provides an impressive inhibition activity of compound **9B** against hBChE by facilitating interactions with CAS residues, which is crucial for hBChE inhibition. Differently, we note a nearly exact placement of the 2-phenyl ring of compounds **10A** and **10B** inside the catalytic subsite, and in this case, the added feature induces a marginally better binding energy value (Table 2). This marginal improvement can be attributed to pi–pi stacking interactions of compound **10B** with residues Trp 82 and Tyr 332 (Figure 1B). Our reported trend of docking energy values between the two compounds (**10A**, **10B**) is consistent with a moderate improvement in experimental IC_50_ values (Table 2). The derivatives’ physicochemical properties, illustrated in Table 3, are a good indicator of their capacity to cross through cellular membranes and predictors of their absorption, distribution, metabolism, and excretion (ADME) parameters.

Compounds with the same halogen substitution of benzofuran moiety (but different in their position) and a hydroxyl group in the para position of the phenyl ring displayed the same physicochemical properties. All the molecules have two rotatable bonds, thus showing similar flexibility.

Theoretically, all the 2-benzylbenzofuran derivatives have acceptable physicochemical properties to display good bioavailability, and none of them violate the Lipinski rule. The studied compounds exhibit appreciable penetration capability across the biological membranes, and no pan-assay interference structures (PAINS) were detected, according to the obtained results (Table 3).

Therefore, based on desirable physicochemical and structural features shown, the investigated compounds exhibit drug-likeness properties.

## 3. Materials and Methods

### 3.1. Chemistry

Under an atmosphere of nitrogen with magnetic stirring, reactions were performed in oven-dried glassware. The reagents and starting materials were purchased from retail suppliers (Merck/Sigma-Aldrich, Darmstadt, Germany) and were used without further purification. Melting points (mp) are uncorrected and determined with a Reichert Kofler thermopan or in capillary tubes in a Büchi 510 apparatus.

^1^H NMR and ^13^C NMR spectra were recorded with a Varian INOVA 500 spectrometer and a Varian INOVA 600 spectrometer, using CDCl_3_ as solvent. Chemical shifts (δ) are expressed in parts per million (ppm) using TMS as an internal standard. Coupling constants *J* are expressed in hertz (Hz). Spin multiplicities are given as s (singlet), d (doublet), dd (doublet of doublets), and m (multiplet). Elemental analyses were performed by using a Perkin Elmer 240B microanalyzer and are within 0.4% of calculated values in all cases. The analytical results indicate 98% purity for all compounds. Flash chromatography (FC) was performed on silica gel (Merck 60, 230–400 mesh); analytical thin-layer chromatography (TLC) was performed on pre-coated silica gel plates (Merck 60 F254), visualized by exposure to UV light. Organic solutions were dried over anhydrous sodium sulphate (Na_2_SO_4_). After reaction or extraction, the solvent’s concentration and evaporation were carried out on a rotary evaporator (Büchi Rotavapor, Cornaredo Italia) operating under reduced pressure.

### 3.2. Determination of Butyrylcholinesterase Inhibition

The cholinesterase inhibition activity was executed using Ellman’s method [22], as previously described with slight modifications [10].

S-Butyrylthiocholine iodide (BTCI) and 5,5′-dithiobis-(2-nitrobenzoic) acid (DTNB) (Merck/Sigma-Aldrich) were used for the determination of the BChE activity. Butyrylcholinesterase (EC 3.1.1.8, BChE) was from equine serum (eqBChE) (Sigma-Aldrich). We used 96-well plates for analysis, to each well we added 70 μL phosphate-buffered (0.1 M, pH 8.0), 4 μL different concentration of the tested compounds or dimethyl sulfoxide (for control experiment), 6 μL enzyme (final concentration 0.15 U/mL in buffer), and 100 μL DTNB (1.5 mM final). The solution was preincubated for 5 min at 37 °C, then 20 μL BTCI 1.5 mM solution as the substrate was added to the reaction mixture. The absorbance change was measured at 405 nm, with a microplate reader FLUOstar OPTIMA (BMG Labtech, Offenburg, Germany). Galantamine was used as a standard inhibitor. Each experiment was done in triplicate. IC_50_ values were calculated as the concentration of compound with 50% inhibitory activity and were obtained from activity (slope) versus compound concentration plots.

The statistical significance of differences was evaluated using one-way analysis of variation (one-way ANOVA), and Tukey’s multiple comparisons. Results were expressed as mean ±  standard deviation (SD), and statistically significant differences were evaluated with *p* < 0.001 as a minimal significance level.

### 3.3. Molecular Modelling Studies

The three-dimensional (3D) protein structure of human BChE was downloaded from the protein data bank (PDB id: 4TPK). The chemical structure of the ligands was drawn and converted into 3D coordinates using online openbabel software (http://www.cheminfo.org/Chemistry/Cheminformatics/FormatConverter/index.html, accessed on 22 February 2022). Geometric optimization of the ligand structure was done using density functional theory formulation of quantum mechanics [23], and it was subsequently converted into mol2 format for the docking experiment. Chain A from of the crystal structure (PDB id: 4TPK) for protein and mol2 format for the ligand were submitted for docking using the SwissDock web server [24]. The docking program is based on EADock dihedral space sampling (DSS) engine [25], which uses a multiobjective scoring function based on CHARMM22 force field and an implicit treatment of the solvent by using a fast analytical continuum solvation model. The docking procedure considers the entire protein surface, and 5000 ligand-binding modes are generated, which were then classified and ranked into different clusters using a scoring function. The predictions file provides the user information on cluster Rank, full fitness, and estimated binding free energy ΔG (kcal/mol) values.

To validate our docking method, we performed a study using a co-crystal ligand (*N*-{[(3*R*)-1-(2,3-dihydro-1*H*-inden-2-yl)piperidin-3-yl]methyl}-*N*-(2-methoxyethyl)naphthalene-2-carboxamide) and the docked structures of the same ligand obtained from Swissdock webserver. The co-crystal structure of the ligand was used as a reference (crystal), and the best docked structure of the same ligand (model) from Swissdock protocol was compared (Supporting Information—2. Molecular modelling studies Figure S1).

The presence of pan-assay interference structures (PAINS) and the conformity to Lipinski’s rule was investigated by employing the free web tool Swiss ADME (http://www.swissadme.ch/, accessed on 22 February 2022) [21].

## 4. Conclusions

Our research endeavors have focused on developing novel 2-phenylbenzofuran derivatives with improved inhibitory activity against the BChE enzyme, a viable target in AD. In the hope of favorably improving the inhibitory action of our previously synthesized derivatives, we introduced a methylene spacer between the benzofuran and the 2-phenyl ring moieties. The present study shows that introducing a methylene spacer in compound **9A** (that showed no BChE inhibition) drastically enhanced the compound’s (**9B**) action against the enzyme. Therefore, the additive structural feature alters the flexibility of the molecule such that it readily nestles and interacts with catalytic triad residues, which is pivotal for enzyme inhibition. Altogether, our results highlighted the importance of late-stage functionalization as a desirable approach to accelerate the drug discovery process against AD.

## Figures and Tables

**Table 1:** Inhibitory effect (IC_50_) of compounds **9**–**16**(**A**) and **9**–**16**(**B**) on BChE activity. The data shown are the mean ± SD of three independent experiments.

Compounds 9–16(B)		Compounds 9–16(A)	
IC_50_ (µM)			
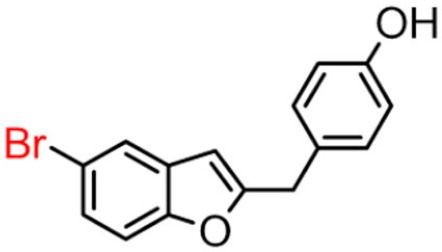 **9B**	2.93 ± 0.38	>100	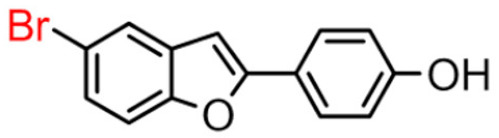 **9A** [8]
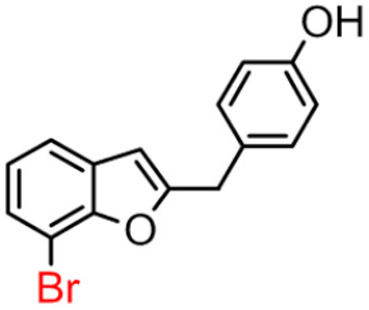 **10B**	32.60 ± 2.04	82.5 ± 7.1	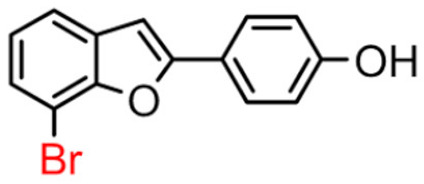 **10A** [8]
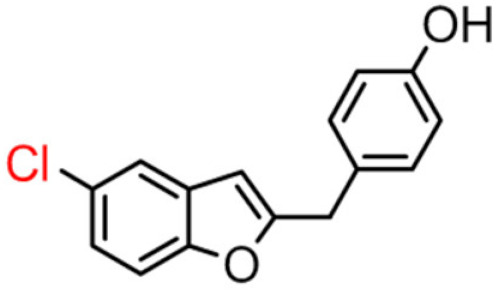 **11B**	55.66 ± 0.80	>100	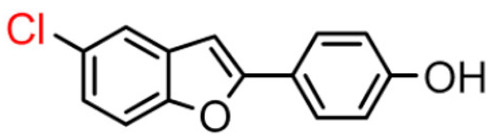 **11A** [8]
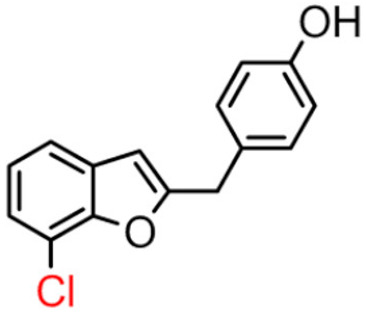 **12B**	42.98 ± 5.65	30.3 ± 2.1	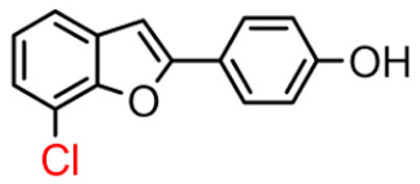 **12A** [8]
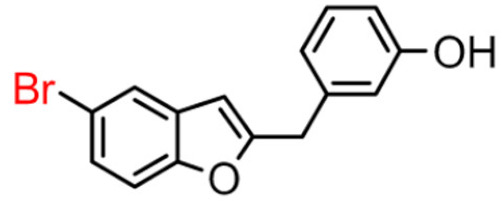 **13B**	39.95 ± 4.31	10.60 ± 1.65	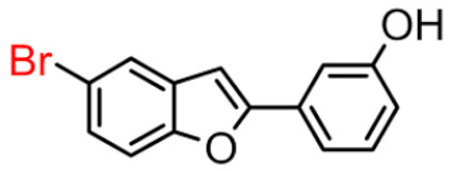 **13A**
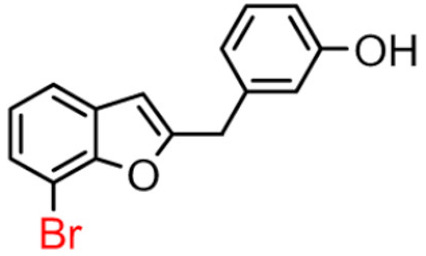 **14B**	31.37 ± 0.15	7.96 ± 0.28	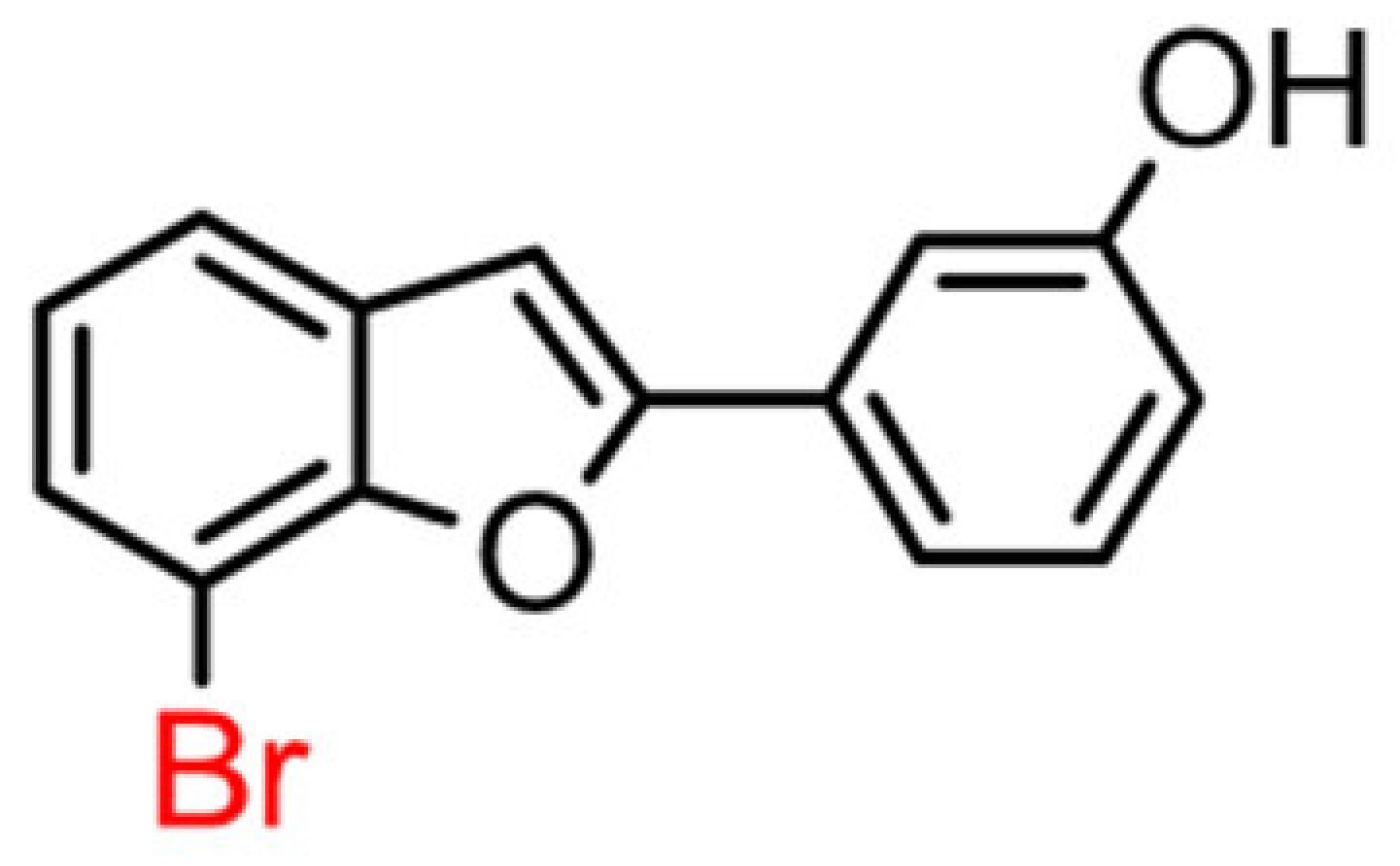 **14A** [10]
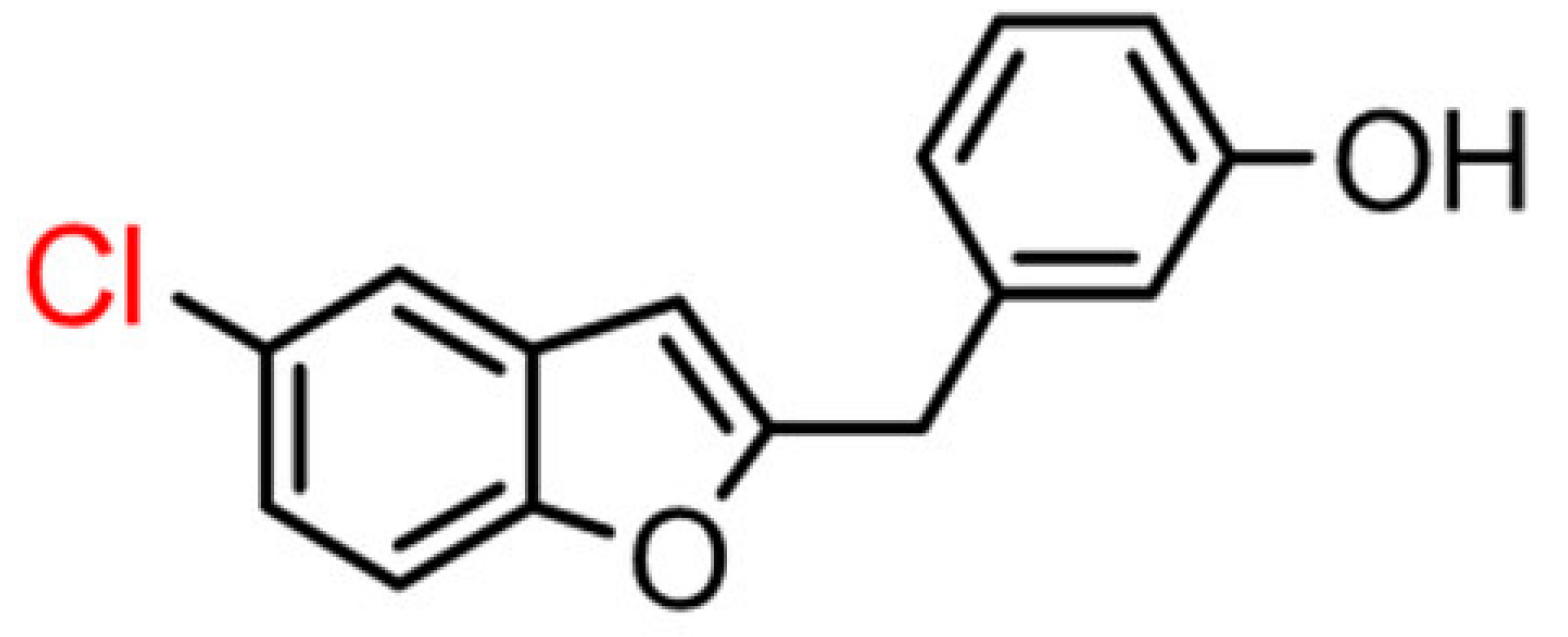 **15B**	27.84 ± 3.07	10.52 ± 0.40	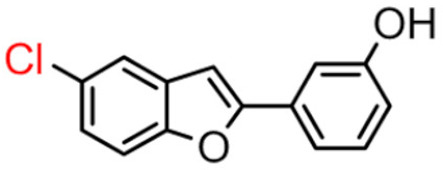 **15A**
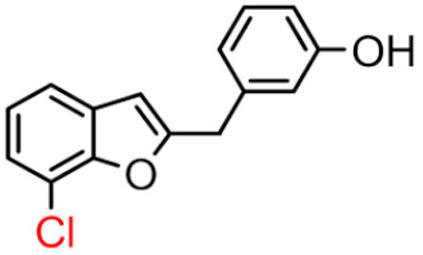 **16B**	61.65 ± 5.90	13.42 ± 2.57	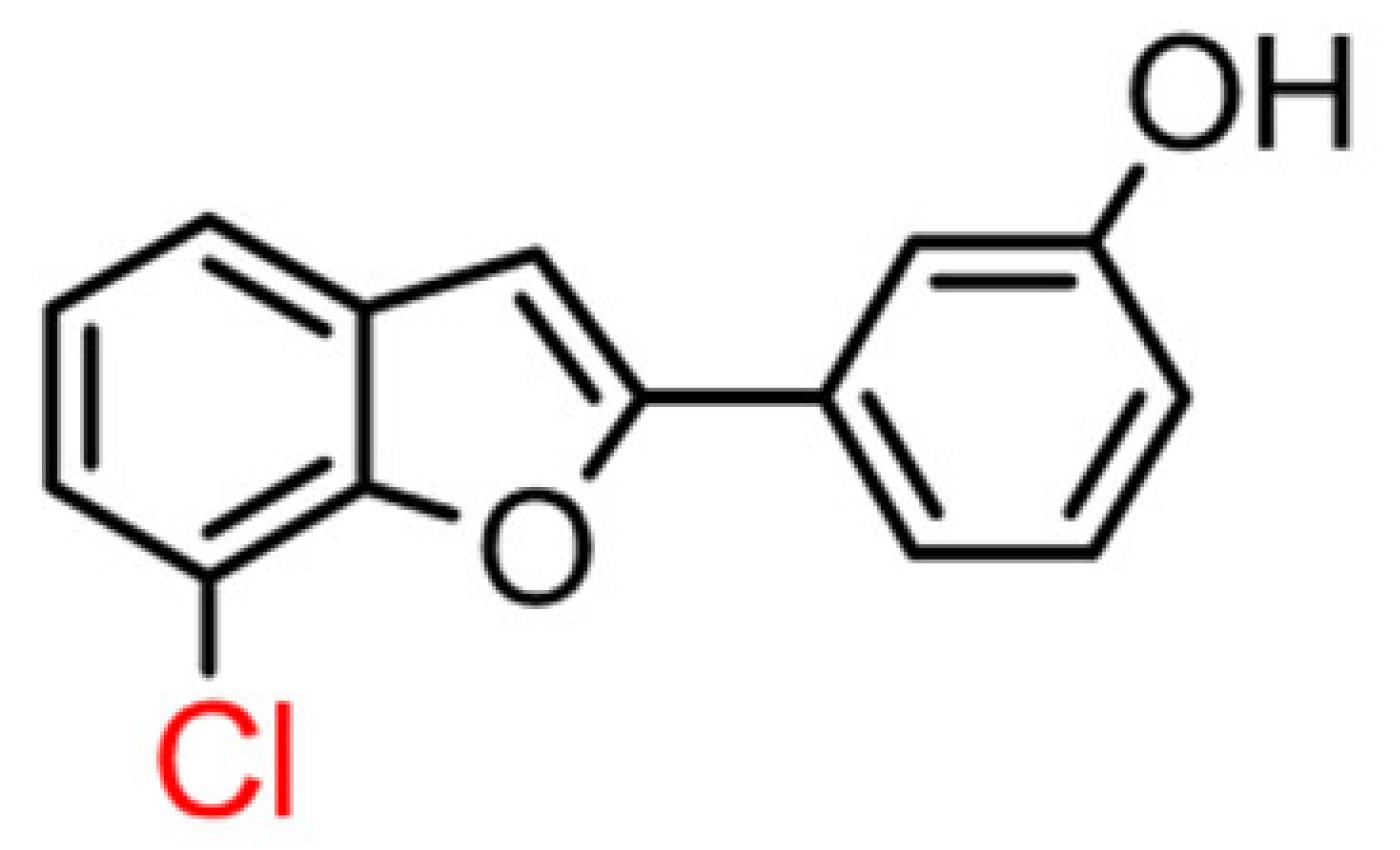 **16A** [10]
**Galantamine**	28.06 ± 2.10		

**Table 2:** Molecular docking energies scores and IC_50_ values of compounds (**9A**, **9B**, **10A**, and **10B**) against hBChE. (Docking energies values for all compounds are reported in Supporting Information—2. Molecular modelling studies Table S1).

Compound	Docking Energy (kcal/mol)	IC_50_ (µM)
**9A**	−7.0	>100
**9B**	−8.8	2.9
**10A**	−7.5	82.5
**10B**	−7.8	32.6

**Table 3:** Molecular properties of **9A**/**10A** and **9B**/**10B** were calculated using the Swiss ADME software [21].

Physicochemical Properties	Compound	
	**Without** **Spacer** **9A/10A**	**With** **Spacer** **9B/10B**
MW (g/mol)	289.12	303.15
Molecular volume (Å^3^)	206.91	223.72
Rotatable bonds	1	2
H-bond acceptor atoms	2	2
H-bond donor atoms	1	1
TPSA (Å^2^)	33.37	33.37
Lipophilicity (logP_o/w_)	3.75/3.73	4.00/3.93
Water solubility	Moderate	Moderate
Drug-likeness (Lipinski)	Yes; 0 viol	Yes; 0 viol
GI absorption	High	High
BBB permeability	Yes	Yes
PAINS	0	0

MW—molecular weight; TPSA—topological polar surface area; Log P—expressed as the logarithm of octanol/water partition coefficient; Drug-likeness—Number of violations of Lipinski’s rules; GI—Gastrointestinal; BBB—blood–brain barrier; PAINS—pan-assay interference structure.

## Data Availability

Data is contained within the article.

## Supplementary Materials

The following are available online at https://www.mdpi.com/article/10.3390/ph15030304/s1, General Experimental Procedure for the Synthesis; ^1^H NMR, ^13^C NMR spectra; Figure S1: Superimposition of the reference complex (PDB code: 4TPK) and the best docked ligand pose obtained from Swissdock webserver; Table S1: Effect of spacer on the docking and IC_50_ values of compounds.
