# Supplementary material for: Structural Insight of New Butyrylcholinesterase Inhibitors Based on Benzylbenzofuran Scaffold
Source: Pharmaceuticals (Basel). 2022 Mar 2;15(3):304. doi: 10.3390/ph15030304 (PMC8955773; doi:10.3390/ph15030304)
Supplement: Supplementary file 1 [file pharmaceuticals-15-00304-s001.zip › pharmaceuticals-1592799-supplementary.pdf]

pharmaceuticals-1592799

Supporting Information

Structural insight of new Butyrylcholinesterase inhibitors based on Benzylbenzofuran scaffold

Giovanna L. Delogu[1], Antonella Fais[1], Francesca Pintus[1], Chinmayi Goyal[2], Maria J. Matos[3], Benedetta Era[1, *] and Amit Kumar[4]

[1]Department of Life and Environmental Sciences, University of Cagliari, Monserrato, Cagliari, 09042, Italy; delogug@unica.it; fais@unica.it; fpintus@unica.it; era@unica.it

[2]Yorktown High School, 2727 Crompond Road, Yorktown Heights, NY 10598, USA; chinmayigoyal.1@gmail.com

[3]Departamento de Quìmica Orgànica, Facultade de Farmacia, Universidade de Santiago de Compostela, 15782 Santiago de Compostela, Spain; mariacmatos@gmail.com

[4]Department of Electrical and Electronic Engineering, University of Cagliari, via Marengo 2, Cagliari, 09123, Italy; amit369@gmail.com or amit.kumar@unica.it

* Correspondence: era@unica.it (B.E).

1. Chemistry

1.1 General Experimental Procedure for the Synthesis of methoxylated 2-phenylbenzofurans **5A** and **7A**

2.1 General Experimental Procedure for the Synthesis of hydroxylated 2-phenylbenzofurans **13A** and **15A**

3.1 General Experimental Procedure for the Synthesis of methoxylated 2-benzylbenzofurans **1-8(B)**

4.1 General Experimental Procedure for the Synthesis of hydroxylated 2-benzylbenzofurans **9-16(B)**

5.1 ^{1}H NMR and ^{13}C NMR spectra 2-phenylbenzofurans hydroxylated **13A** and **15A**

6.1 ^{1}H NMR and ^{13}C NMR spectra 2-benzylbenzofurans hydroxylated **9-16(B)**

2. Molecular modelling studies

2.1 Docking studies

Table S.2.1 Table S1

2.2 Validation of Docking Method

Figure S.2.1. Figure S1

1. Chemistry

1.1 General Experimental Procedure for the Synthesis of methoxylated 2-phenylbenzofurans 1-8(A)

A mixture of 2-hydroxybenzyltriphenylphosphonium bromide **IIa-IId** (0.50 g, 1.11 mmol) and benzoyl chloride (0.12 mL, 1.11 mol) in a mixed solvent (toluene 20 mL and Et_3N 0.5 mL) was stirred under reflux for 2h. The precipitate was removed by filtration. The filtrate was concentrated, and the residue was purified by silica gel chromatography (hexane/EtOAc 9:1) to give the desired compounds **1–8(A)** [8,10].

*5-bromo-2-(3-methoxyphenyl) benzofuran **5A***. Yield: 75%; m.p.: 93-94 °C; ^{1}H NMR (500 MHz, $CDCl_3$), *δ* (ppm), *J* (Hz) = 3.90 (s, 3H, OCH_3), 6.91-6.94 (m, 2H), 7.34-7.44 (m, 5H), 7.70 (s, 1H); ^{13}C NMR (125 MHz, $CDCl_3$), *δ* (ppm), *J* (Hz) = 55.53, 101.28, 110.84, 112.74, 115.04, 116.15, 117.80, 123.63, 127.29, 130.07, 131.31, 131.32, 153.73, 157.21, 160.13; Anal. calcd. for $C_{15}H_{11}BrO_2$: C, 59.43%; H, 3.66%. Found: C, 59.48%; H, 3.70%.

*5-chloro-2-(3-methoxyphenyl) benzofuran **7A***. Yield: 78%; m.p.: 64-66 °C; ^{1}H NMR (500 MHz, $CDCl_3$), *δ* (ppm), *J* (Hz) = 3.89 (s, 3H, OCH_3), 6.93-6.95 (m, 1H), 6.96 (s, 1H), 7.30-7.32 (m, 2H), 7.24 (d, *J* = 8.7 Hz, 1H), 7.41-7.46 (m, 2H), 7.52-7.56 (m, 1H); ^{13}C NMR (125 MHz, $CDCl_3$), *δ* (ppm), *J* (Hz) = 55.55, 101.26, 110.48, 112.27, 115.04, 117.80, 120.59, 124.61, 128.67, 130.09, 130.69, 131.40, 153.38, 157.39, 160.14; Anal. calcd. for $C_{15}H_{11}ClO_2$: C, 69.64%; H, 4.29%. Found: C, 69.70%; H, 4.33%.

2.1 General Experimental Procedure for the Synthesis of hydroxylated 2-phenylbenzofurans 9-16(A)

A solution of the methoxy-2-phenylbenzofuran (0.11 g, 0.050 mmol) in acetic acid (5.0 mL) and acetic anhydride (5.0 mL), at 0°C, was prepared. Hydriodic acid 57% (10.0 mL) was added dropwise. The mixture was stirred under reflux temperature for 3h. The solvent was evaporated under vacuum, and the dry residue was purified by FC (dichloromethane/methanol 9.8:0.2) to give the desired compound **9-16(A)** [8,10,20].

*5-bromo-2-(3-hydroxyphenyl)benzofuran **13A***. Yield: 65%; m.p.: 127-129°C; ^{1}H NMR (500 MHz, $CDCl_3$), *δ* (ppm), *J* (Hz) = 6.85 (s, 1H); 7.29-7.32 (m, 3H), 7.35-7.36 (m, 1H), 7.43 (dd *J* = 8.7, 2.0 Hz, 1H), 7.61 (d, *J* = 8.7 Hz, 1H), 7.84 (d, *J* = 1.9 Hz, 1H), 9.72 (s, 1H, -OH); ^{13}C NMR (125 MHz, $CDCl_3$), *δ* (ppm), *J* (Hz) = 101.26, 110.04, 115.04, 117.80, 120.59, 124.61, 128.67, 130.09, 130.69, 131.40, 153.38, 157.39, 160.14; Anal. calcd**.** for $C_{14}H_9BrO_2$: C,58.16%; H, 3.14%. Found: C, 58.24%; H, 3.25%.

5-chloro-2-(3-hydroxyphenyl)benzofuran ***15A***. Yield: 78%; m.p.: 115-157 °C; ^{1}H NMR (500 MHz, $CDCl_3$), δ (ppm), J (Hz) = 6.86 (s, 1H), 7.29-7.35 (m, 4H), 7.36 (d, J = 8.7 Hz, 1H), 7.66 (d, J = 8.4 Hz, 1H), 7.70 (d, J = 2.1 Hz, 1H), 9.73 (s, 1H, -OH); ^{13}C NMR (125 MHz, $CDCl_3$), δ (ppm), J (Hz) = 101.39, 111.99, 112.28, 116.19, 117.94, 120.64, 124.70, 128.70, 130.35, 134.94, 153.31, 156.05, 157.06; Anal. calcd. for $C_{14}H_9ClO_2$: C, 68.72%; H, 3.71%. Found: C, 68.76%; H, 3.80%.

3.1 General Experimental Procedure for the Synthesis of methoxylated 2-benzylbenzofurans 1-8(B)

2-Hydroxybenzyltriphenylphosphonium bromide **IIa-IId** (1 g, 2.22 mmol) and 2-phenylacetyl chloride (0.32 mL, 2.22 mmol) in a mixed solvent (toluene 50 mL and Et_3N 1mL) were heated to reflux for 2h. The mixture is allowed to cool, and the white precipitate formed is filtered off. The filtrate is evaporated to dryness, and the residue is purified by silica gel chromatography (hexane/EtOAc 9:1) to obtain the desired compounds **1–8(B)**.

5-Bromo-2-(4-methoxybenzyl)benzofuran ***1B***. Yield: 32 %; m.p.: brown oil; ^{1}H NMR (600 MHz, $CDCl_3$), δ (ppm), J (Hz) = 3.72 (s, 3H, OCH_3), 3.95 (s, 2H, CH_2), 6.21 (s, 1H), 6.79 (d, J = 8.6 Hz, 2H), 7.13 (d, J = 8.6 Hz, 2H), 7.16-7.23 (m, 2H), 7.49 (d, J = 1.9 Hz, 1H) ppm; ^{13}C NMR (150 MHz, $CDCl_3$), δ (ppm), J (Hz) = 34.26, 55.43, 102.80, 112.47, 114.25, 115.69, 123.18, 126.35, 128.91, 130.05, 131.00, 153.86, 158.72, 159.97 ppm; Anal. calcd for $C_{16}H_{13}BrO_2$: C, 60.59%; H, 4.13%. Found: C, 60.64%; H, 4.18%.

7-Bromo-2-(4-methoxybenzyl)benzofuran ***2B***. Yield: 35 %; m.p.: brown oil; ^{1}H NMR (600 MHz, $CDCl_3$), δ (ppm), J (Hz) = 3.72 (s, 3H, OCH_3), 4.00 (s, 2H, CH_2), 6.25 (s, 1H), 6.80 (d, J = 8.6 Hz, 2H), 6.96 (t, J = 7.7 Hz, 1H), 7.15 (d, J = 8.6 Hz, 2H), 7.27-7.30 (m, 2H) ppm; ^{13}C NMR (150 MHz, $CDCl_3$), δ (ppm), J (Hz) = 34.21, 55.42, 103.73, 104.02, 114.23, 119.68, 123.94, 126.59, 128.87, 130.17, 130.28, 152.15, 158.72, 159.73 ppm; Anal. calcd for $C_{16}H_{13}BrO_2$: C, 60.59%; H, 4.13%. Found: C, 60.61%; H, 4.16%.

5-Chloro-2-(4-methoxybenzyl)benzofuran ***3B***. Yield: 30 %; m.p.: yellow oil; ^{1}H NMR (600 MHz, $CDCl_3$), δ (ppm), J (Hz) = 3.80 (s, 3H, OCH_3), 4.03 (s, 2H, CH_2), 6.29 (s, 1H), 6.88 (d, J = 8.7 Hz, 2H), 7.15 (dd, J = 8.7, 2.1 Hz, 1H), 7.21 (d, J = 8.6 Hz, 2H), 7.30 (d, J = 8.7 Hz, 1H), 7.42 (d, J = 2.1 Hz, 1H) ppm; ^{13}C NMR (150 MHz, $CDCl_3$), δ (ppm), J (Hz) = 34.29, 55.42, 102.94, 111.95, 114.24, 120.13, 123.63, 128.93, 130.05, 130.36, 132.19, 153.48, 158.71, 160.12 ppm; Anal. calcd for $C_{16}H_{13}ClO_2$: C, 70.46%; H, 4.80%. Found: C, 70.48%; H, 4.84%.

7-Chloro-2-(4-methoxybenzyl)benzofuran ***4B***. Yield: 30 %; m.p.: brown oil; ^{1}H NMR (600 MHz, $CDCl_3$), δ (ppm), J (Hz) = 3.84 (s, 3H, OCH_3), 4.12 (s, 2H, CH_2), 6.35 (s, 1H), 6.92 (d, J = 8.6 Hz, 2H), 7.13 (t, J = 7.8 Hz, 1H), 7.24 (dd, J = 7.8, 0.9 Hz, 1H), 7.27 (d, J = 8.6 Hz, 2H), 7.37 (dd, J = 7.7, 0.9 Hz, 1H) ppm;.^{13}C

NMR (150 MHz, $CDCl_3$), *δ* (ppm), *J* (Hz) = 34.19, 55.41, 103.88, 114.23, 116.39, 119.05, 123.54, 123.73, 128.87, 130.15, 130.61, 150.78, 158.71, 159.77 ppm; Anal. calcd for $C_{16}H_{13}ClO_2$: C, 70.46%; H, 4.80%. Found: C, 70.47%; H, 4.83%.

5-Bromo-2-(3-methoxybenzyl)benzofuran ***5B***. Yield: 35 %; m.p.: yellow oil; ^1H NMR (600 MHz, $CDCl_3$), *δ* (ppm), *J* (Hz) = 3.70 (s, 3H, OCH_3), 3.97 (s, 2H, CH_2), 6.24 (s, 1H), 6.70-6.76 (m, 2H), 6.79 (d, *J* = 7.6 Hz, 1H), 7.13-7.18 (m, 2H), 7.21 (dd, *J* = 8.7, 1.9 Hz, 1H), 7.49 (d, *J* = 1.9 Hz, 1H) ppm; ^{13}C NMR (150 MHz, $CDCl_3$), *δ* (ppm), *J* (Hz) = 35.10, 55.32, 103.08, 112.33, 112.48, 114.89, 115.71, 121.38, 123.21, 126.42, 129.80, 130.94, 138.40, 153.84, 159.25, 159.98 ppm; Anal. calcd for $C_{16}H_{13}BrO_2$: C, 60.59%; H, 4.13%. Found: C, 60.63%; H, 4.16%.

7-Bromo-2-(3-methoxybenzyl)benzofuran ***6B***. Yield: 40 %; m.p.: yellow oil; ^1H NMR (600 MHz, $CDCl_3$), *δ* (ppm), *J* (Hz) = 3.72 (s, 3H, OCH_3), 4.03 (s, 2H, CH_2), 6.30 (s, 1H), 6.73 (dd, *J* = 8.2, 2.4 Hz, 1H), 6.78-6.84 (m, 2H), 6.93-6.98 (m, 1H), 7.17-7.21 (m, 2H), 7.30 (m, 1H) ppm; ^{13}C NMR (150 MHz, $CDCl_3$), *δ* (ppm), *J* (Hz) = 35.06, 55.34, 103.75, 104.27, 112.46, 114.89, 119.73, 121.49, 123.97, 126.66, 129.78, 130.23, 138.35, 152.15, 158.98, 159.98 ppm; Anal. calcd for $C_{16}H_{13}BrO_2$: C, 60.59%; H, 4.13%. Found: C, 60.65%; H, 4.19%.

5-Chloro-2-(3-methoxybenzyl)benzofuran ***7B***. Yield: 45 %; m.p.: yellow oil °C; ^1H NMR (600 MHz, $CDCl_3$), *δ* (ppm), *J* (Hz) = 3.83 (s, 3H, OCH_3), 4.10 (s, 2H, CH_2), 6.38 (s, 1H, H-3), 6.84-6.90 (m, 2H, H-3’, H-5'), 6.91-6.95 (m, 1H, H-7’), 7.20 (dd, *J* = 8.7, 2.2 Hz, 1H, H-6), 7.30 (t, *J* = 7.9 Hz, 1H, H-6’), 7.35 (d, *J* = 8.7 Hz, 1H, H-7), 7.47 (d, *J* = 2.1 Hz, 1H, H-4 ppm; ^{13}C NMR (150 MHz, $CDCl_3$), *δ* (ppm), *J* (Hz) = 35.10, 55.30, 103.21, 111.96, 112.30, 114.89, 120.15, 121.37, 123.69, 128.19, 129.78, 130.30, 138.41, 153.46, 159.39, 159.97 ppm; Anal. calcd for $C_{16}H_{13}ClO_2$: C, 70.46%; H, 4.80%. Found: C, 70.51%; H, 4.87%.

7-Chloro-2-(3-methoxybenzyl)benzofuran ***8B***. Yield: 42 %; m.p.: yellow oil; ^1H NMR (600 MHz, $CDCl_3$), *δ* (ppm), *J* (Hz) = 3.71 (s, 3H, OCH_3), 4.02 (s, 2H, CH_2), 6.28 (s, 1H, H-3), 6.73 (dd, *J* = 8.2, 2.3 Hz, 1H, H-6'), 6.77-6.84 (m, 2H, H-3’, H-5’), 7.00 (t, *J* = 7.8 Hz, 1H, H-7’), 7.12-7.16 (m, 2H, H-5, H-6), 7.23-7.28 (m, 1H, H-4) ppm; ^{13}C NMR (150 MHz, $CDCl_3$), *δ* (ppm), *J* (Hz) = 35.04, 55.33, 104.15, 112.42, 114.90, 116.41, 119.09, 121.48, 123.58, 123.80, 129.78, 130.57, 138.36, 150.79, 159.03, 159.97 ppm; Anal. calcd for $C_{16}H_{13}ClO_2$: C, 70.46%; H, 4.80%. Found: C, 70.50%; H, 4.86%. Anal. calcd for $C_{16}H_{13}ClO_2$: C, 70.46%; H, 4.80%. Found: C, 70.50%; H, 4.86%.

4.1 General Experimental Procedure for the Synthesis of hydroxylated 2-benzylbenzofurans 9-16(B)

General procedure for the preparation of hydroxylated 2-benzylbenzofuran **9-16(B)**.

Methoxy-2-benzylbenzofuran (0.40 g, 1.2 mmol) was dissolved in acetic acid (20 mL) and acetic anhydride (20 mL) and cooled to 0 °C. To this solution, hydriodic acid was added dropwise, and then the reaction mixture was refluxed for 3h. After completion of the reaction, the mixture was filtered, and the solvent was removed to give a crude solid product. Purification was carried out by column chromatography using dichloromethane/methanol 9.8:0.2 to provide the desired compounds 9-16(B).

5-Bromo-2-(4-hydroxybenzyl)benzofuran ***9B****.* Yield: 61 %; m.p.: 83-85 °C; ^{1}H NMR (600 MHz, $CDCl_3$), *δ* (ppm), *J* (Hz) = 4.07 (s, 2H, CH_2), 6.37 (s, 1H), 6.75-6.78 (m, 2H), 6.89 (d, *J* = 8.5 Hz, 1H), 7.23 (t, *J* = 8.7 Hz, 1H), 7.29 (s, 1H), 7.30 (s, 1H, -OH), 7.32-7.34 (m, 1H), 7.62 (d, *J* = 1.9 Hz, 1H) ppm; ^{13}C NMR (150 MHz, $CDCl_3$), *δ* (ppm), *J* (Hz) = 34.71, 103.15, 111.92, 113.74, 115.87, 120.38, 121.34, 123.24, 127.99, 129.88, 138.65, 153.48, 155.89, 159.20 ppm; Anal. calcd for $C_{15}H_{11}BrO_2$: C, 59.43%; H, 3.66%. Found: C, 59.46%; H, 3.69%.

7-Bromo-2-(4-hydroxybenzyl)benzofuran ***10B****.* Yield: 71 %; m.p.: brown oil; ^{1}H NMR (600 MHz, $CDCl_3$), *δ* (ppm), *J* (Hz) = 4.07 (s, 2H, CH_2), 6.34 (s, 1H), 6.82 (d, *J* = 8.5 Hz, 2H), 7.05 (t, *J* = 7.7 Hz, 1H), 7.19 (d, *J* = 8.5 Hz, 2H), 7.26 (s, 1H, -OH), 7.36-7.39 (m, 2H) ppm; ^{13}C NMR (150 MHz, $CDCl_3$), *δ* (ppm), *J* (Hz) = 35.16, 103.76, 104.37, 114.20, 115.86, 119.41, 122.03, 124.64, 126.76, 129.86, 138.66, 152.15, 156.86 158.76 ppm; Anal. calcd for $C_{15}H_{11}BrO_2$: C, 59.43%; H, 3.66%. Found: C, 59.48%; H, 3.71%.

5-Chloro-2-(4-hydroxybenzyl)benzofuran ***11B****.* Yield: 61 %; m.p.: 74-76 °C; ^{1}H NMR (600 MHz, $CDCl_3$), *δ* (ppm), *J* (Hz) = 4.02 (s, 2H, CH_2), 6.29 (s, 1H), 6.80 (d, *J* = 8.5 Hz, 2H), 7.15-7.17 (m, 3H), 7.26 (s, 1H, OH), 7.29 (d, *J* = 8.7 Hz, 1H), 7.42 (d, *J* = 2.1 Hz, 1H) ppm; ^{13}C NMR (150 MHz, $CDCl_3$), *δ* (ppm), *J* (Hz) = 34.92, 103.30, 112.00, 114.04, 115.95, 120.19, 121.51, 123.77, 128.25, 130.04, 130.28, 138.78, 153.48, 155.89, 159.20 ppm; Anal. calcd for $C_{15}H_{11}ClO_2$: C, 69.74%; H, 4.29%. Found: C, 69.77%; H, 4.32%.

7-Chloro-2-(4-hydroxybenzyl)benzofuran ***12B****.* Yield: 99 %; m.p.: brown oil; ^{1}H NMR (600 MHz, $CDCl_3$), *δ* (ppm), *J* (Hz) = 4.08 (s, 2H, CH_2), 6.34 (s, 1H), 6.84 (d, *J* = 8.5 Hz, 2H), 7.13 (t, *J* = 7.8 Hz, 1H), 7.19-7.24 (m, 3H), 7.27 (s, 1H, OH) 7.35-7.37 (m, 1H) ppm;.^{13}C NMR (150 MHz, $CDCl_3$), *δ* (ppm), *J* (Hz) = 34.83, 104.26, 114.04, 116.03, 119.13, 121.63, 123.63, 123.87, 130.05, , 138.72, 150.80, 155.87, 158.83 ppm; Anal. calcd for $C_{15}H_{11}ClO_2$: C, 69.74%; H, 4.29%. Found: C, 69.76%; H, 4.33%.

5-Bromo-2-(3-hydroxybenzyl)benzofuran ***13B***. Yield: 70 %; m.p.: 76-78 °C; ^{1}H NMR (600 MHz, $CDCl_3$), *δ* (ppm), *J* (Hz) = 4.07 (s, 2H, CH_2), 6.37 (s, 1H), 6.75-6.78 (m, 2H), 6.89 (d, *J* = 7.6 Hz, 1H), 7.23 (t, *J* = 7.8 Hz, 1H), 7.29 (t, *J* = 7.8 Hz, 1H), 7.30 (s, 1H, -OH), 7.34 (dd, *J* = 8.7, 1.9 Hz, 1H), 7.62 (d, *J* = 1.9 Hz, 1H) ppm; ^{13}C NMR (150 MHz, $CDCl_3$), *δ* (ppm), *J* (Hz) = 34.90, 103.17, 112.52, 114.04, 115.76, 115.94, 121.53, 123.25, 126.49, 130.06, 130.92, 138.77, 153.87, 155.88, 159.05 ppm; Anal. calcd for $C_{15}H_{11}BrO_2$: C, 59.43%; H, 3.66%. Found: C, 59.49%; H, 3.72%.

7-Bromo-2-(3-hydroxybenzyl)benzofuran ***14B***. Yield: 94 %; m.p.: brown oil; ^{1}H NMR (600 MHz, $CDCl_3$), *δ* (ppm), *J* (Hz) = 4.12 (s, 2H, CH_2), 6.43 (s, 1H), 6.78 (dd, *J* = 8.0, 2.1 Hz, 1H), 6.82 (m, 1H), 6.91-6.93 (m, 1H), 7.08 (t, *J* = 7.6 Hz, 1H), 7.23 (t, *J* = 7.8 Hz, 1H), 7.29 (s, 1H, -OH), 7.40-7.42 (m, 2H) ppm; ^{13}C NMR (150 MHz, $CDCl_3$), *δ* (ppm), *J* (Hz) = 34.83, 103.76, 104.37, 114.04, 116.05, 119.75, 121.63, 124.01, 126.71, 130.04, 130.21, 138.69, 152.15, 155.86, 158.78 ppm; Anal. calcd for $C_{15}H_{11}BrO_2$: C, 59.43%; H, 3.66%. Found: C, 59.47%; H, 3.70%.

5-Chloro-2-(3-hydroxybenzyl)benzofuran ***15B***. Yield: 97 %; m.p.: 60-62 °C; ^{1}H NMR (600 MHz, $CDCl_3$), *δ* (ppm), *J* (Hz) = 4.01 (s, 2H, CH_2), 6.30 (s, 1H), 6.65-6.70 (m, 2H), 6.82 (d, *J* = 7.6 Hz, 1H), 7.02 (d, *J* = 8.7 Hz, 1H), 7.11-7.14 (m, 2H), 7.17 (s, 1H, OH), 7.28 (d, *J* = 2.1 Hz, 1H) ppm; ^{13}C NMR (150 MHz, $CDCl_3$), *δ* (ppm), *J* (Hz) = 34.92, 103.30, 112.00, 114.04, 115.95, 120.19, 121.51, 123.77, 128.25, 130.04, 130.28, 138.78, 153.48, 155.89, 159.20 ppm; Anal. calcd for $C_{15}H_{11}ClO_2$: C, 69.74%; H, 4.29%. Found: C, 69.79%; H, 4.36%.

7-Chloro-2-(3-hydroxybenzyl)benzofuran ***16B***. Yield: 93 %;. m.p.: 78-80 °C; ^{1}H NMR (600 MHz, $CDCl_3$), *δ* (ppm), *J* (Hz) = 4.01 (s, 2H, CH_2), 6.30 (s, 1H), 6.65-6.70 (m, 2H), 6.82 (dd, *J* = 8.1, 2.4 Hz, 1H), 7.02 (t, *J* = 7.8 Hz, 1H), 7.11-7.14 (m, 2H), 7.17 (s, 1H, -OH), 7.28 (dd, *J* = 7.7, 0.9 Hz, 1H) ppm;.^{13}C NMR (150 MHz, $CDCl_3$), *δ* (ppm), *J* (Hz) = 34.83, 104.26, 114.04, 116.03, 116.44, 119.13, 121.63, 123.63, 123.87, 130.05, 130.55, 138.72, 150.80, 155.87, 158.83 ppm; Anal. calcd for $C_{15}H_{11}ClO_2$: C, 69.74%; H, 4.29%. Found: C, 69.78%; H, 4.36%.

5.1 ^{1}H NMR spectra of *5-Bromo-2-(3-hydroxyphenyl)benzofuran* ***13A***

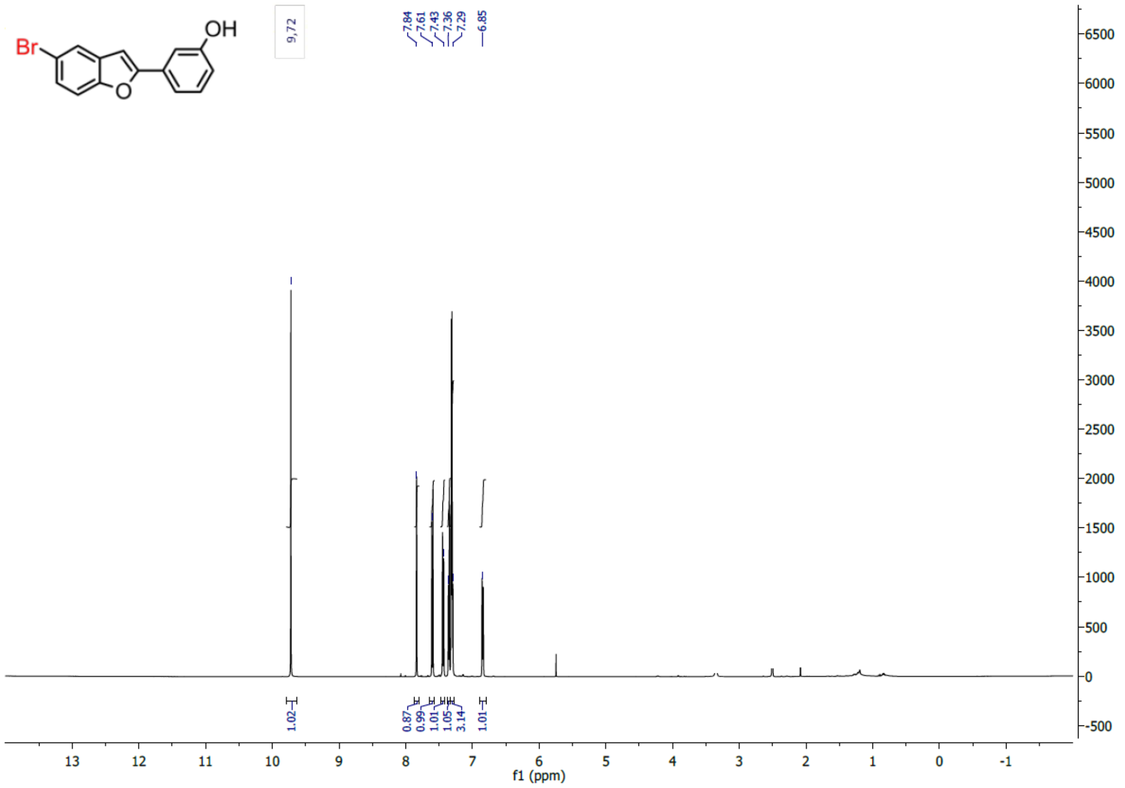

^{13} CNMR spectra of *5-Bromo-2-(3-hydroxyphenyl)benzofuran* ***13A***

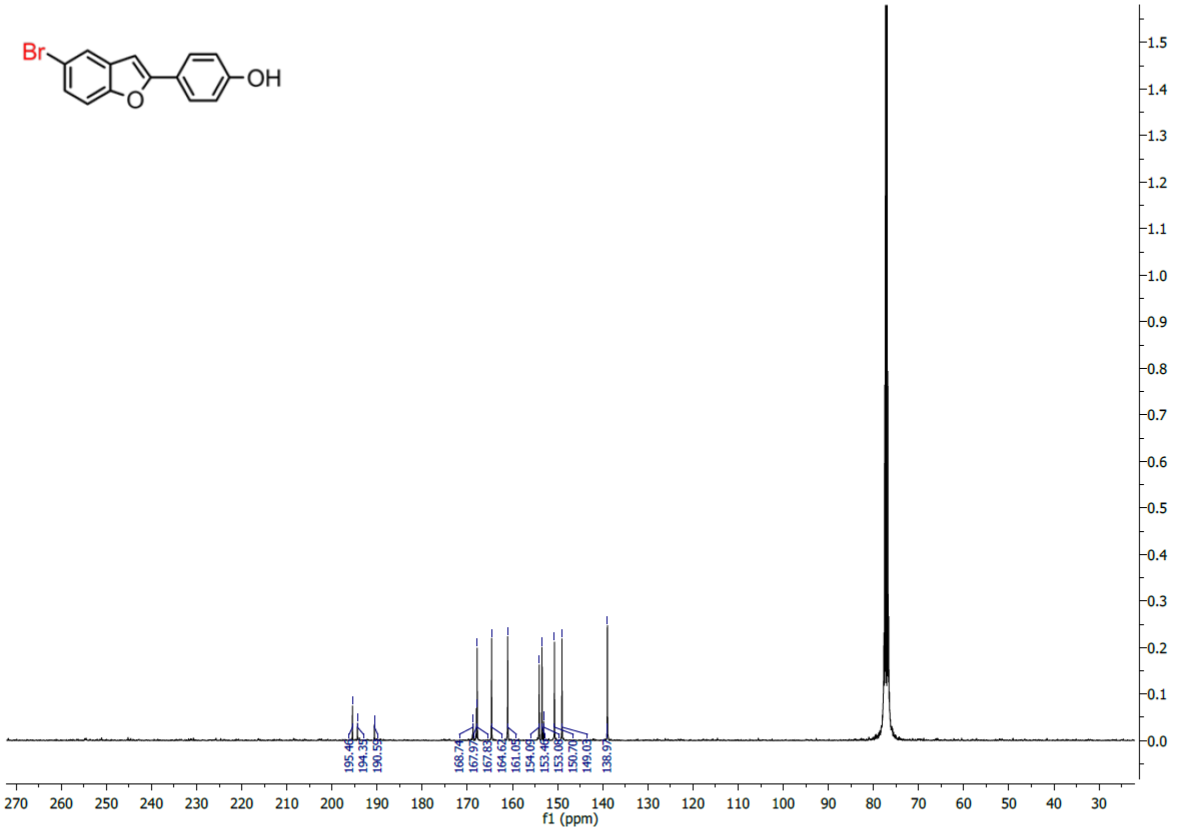

^{1}H NMR spectra of *5-Chloro-2-(3-hydroxyphenyl)benzofuran* ***15A***

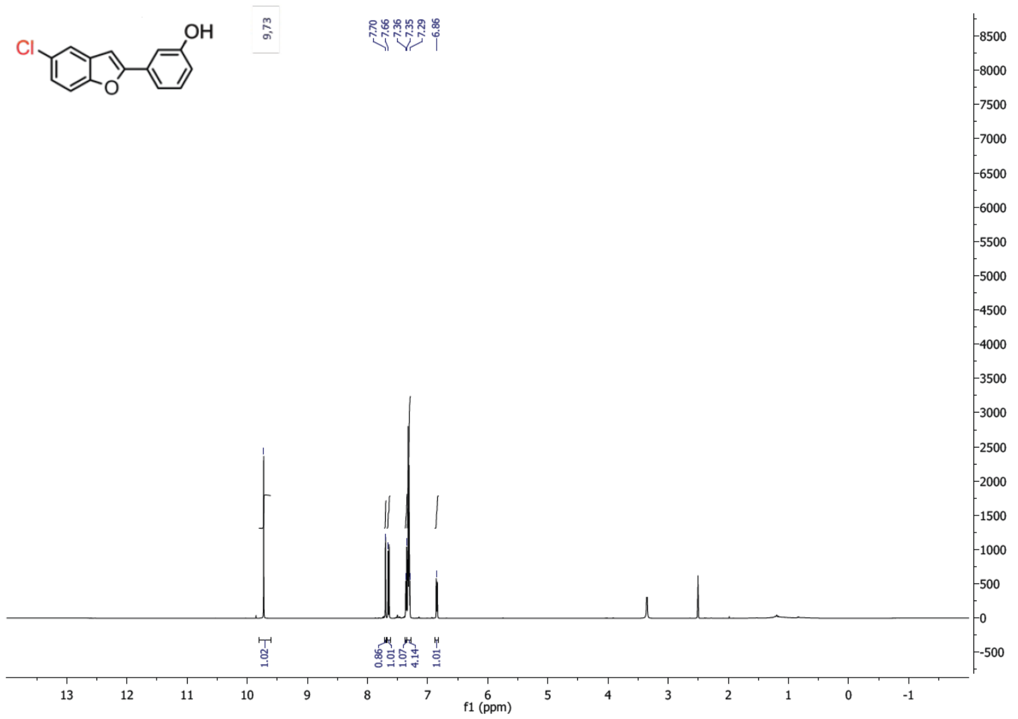

[13] CNMR spectra of *5-Chloro-2-(3-hydroxyphenyl)benzofuran* ***15A***

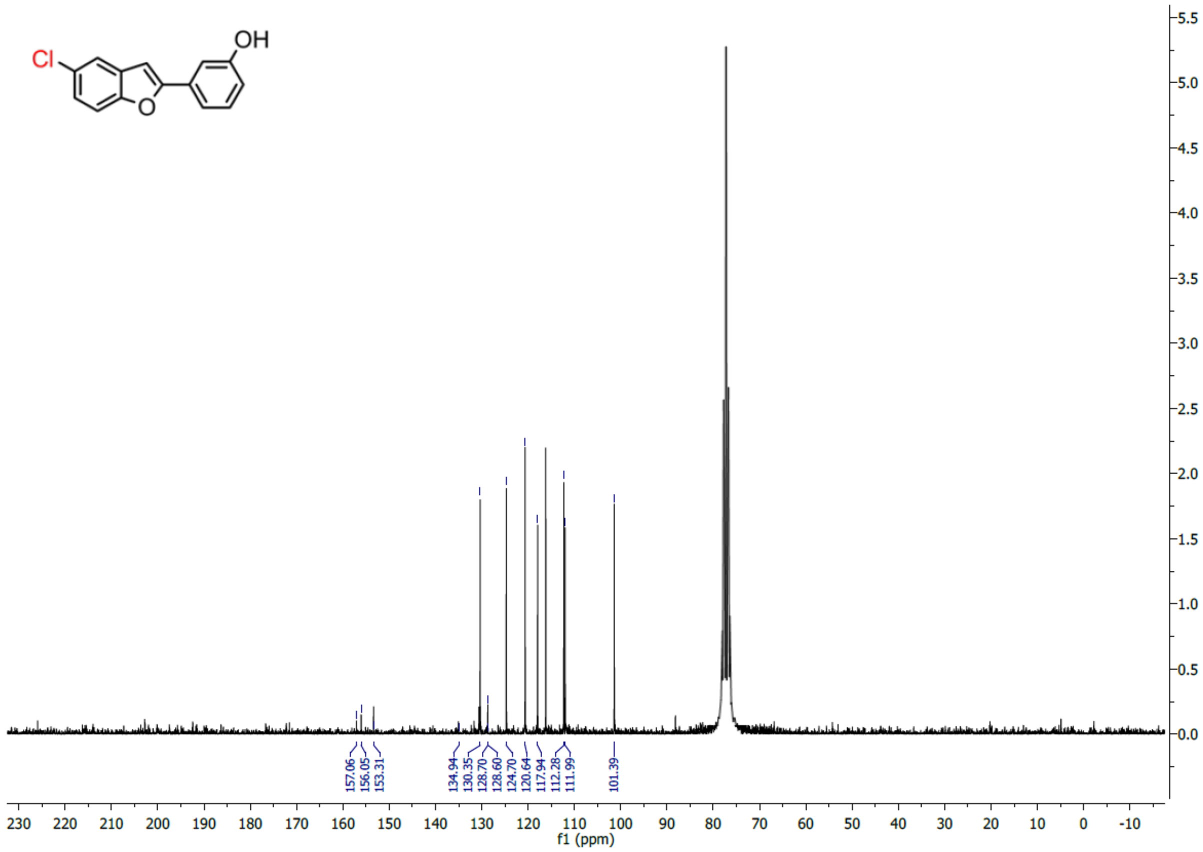

6.1 ^{1}H NMR spectra of *5-Bromo-2-(4-hydroxybenzyl)benzofuran* ***9B***

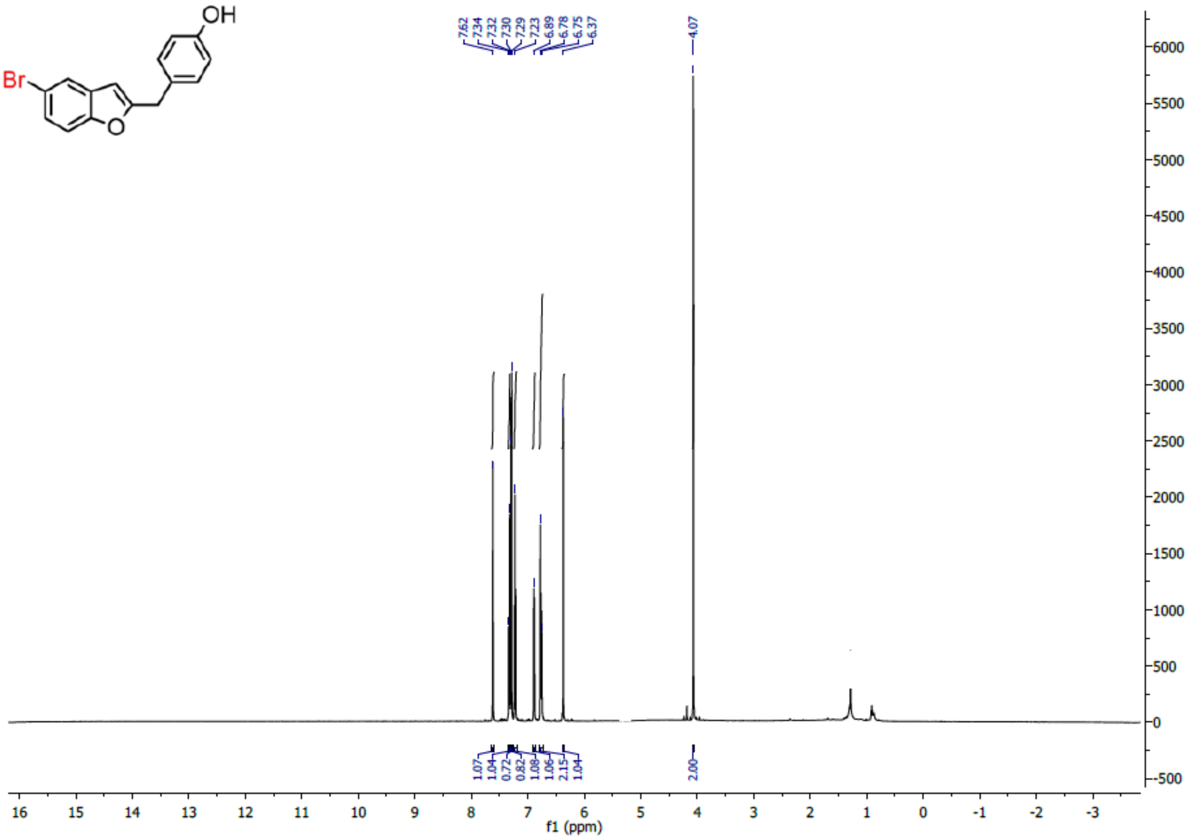

[13] CNMR spectra of *5-Bromo-2-(4-hydroxybenzyl)benzofuran* ***9B***

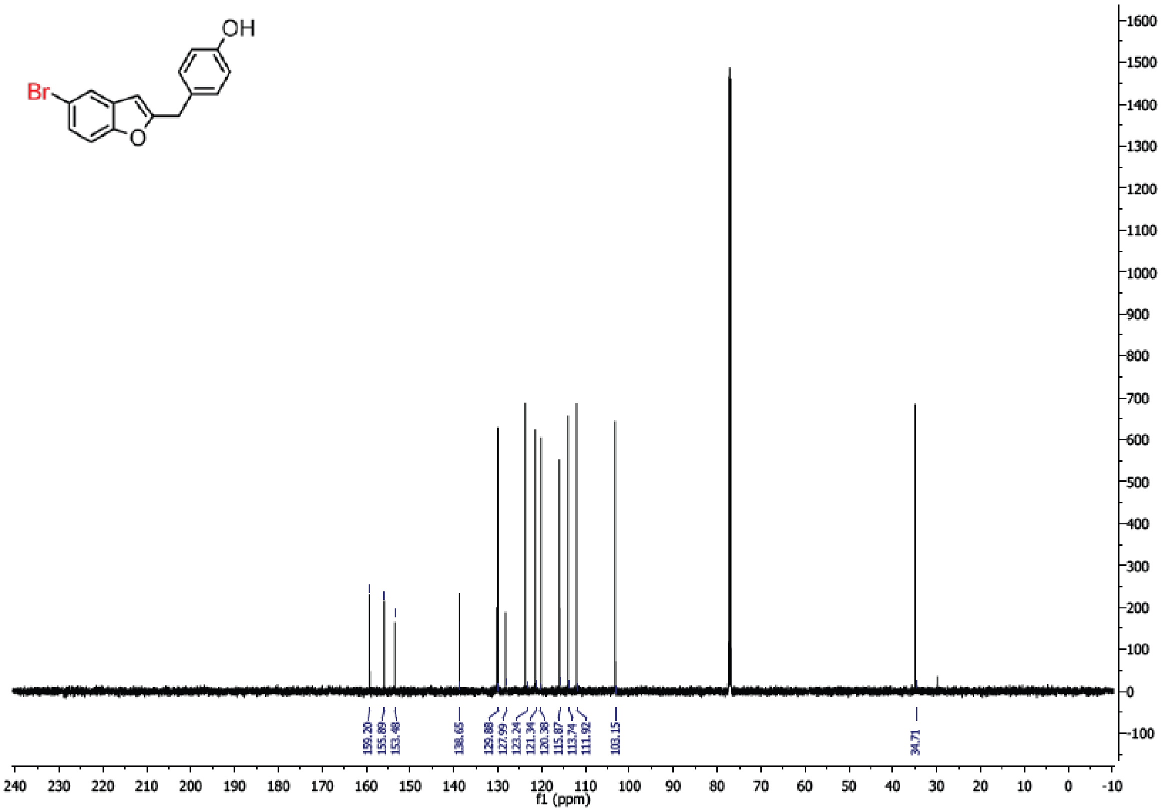

^{1}H NMR spectra of *5-Bromo-2-(4-hydroxybenzyl)benzofuran* ***10B***

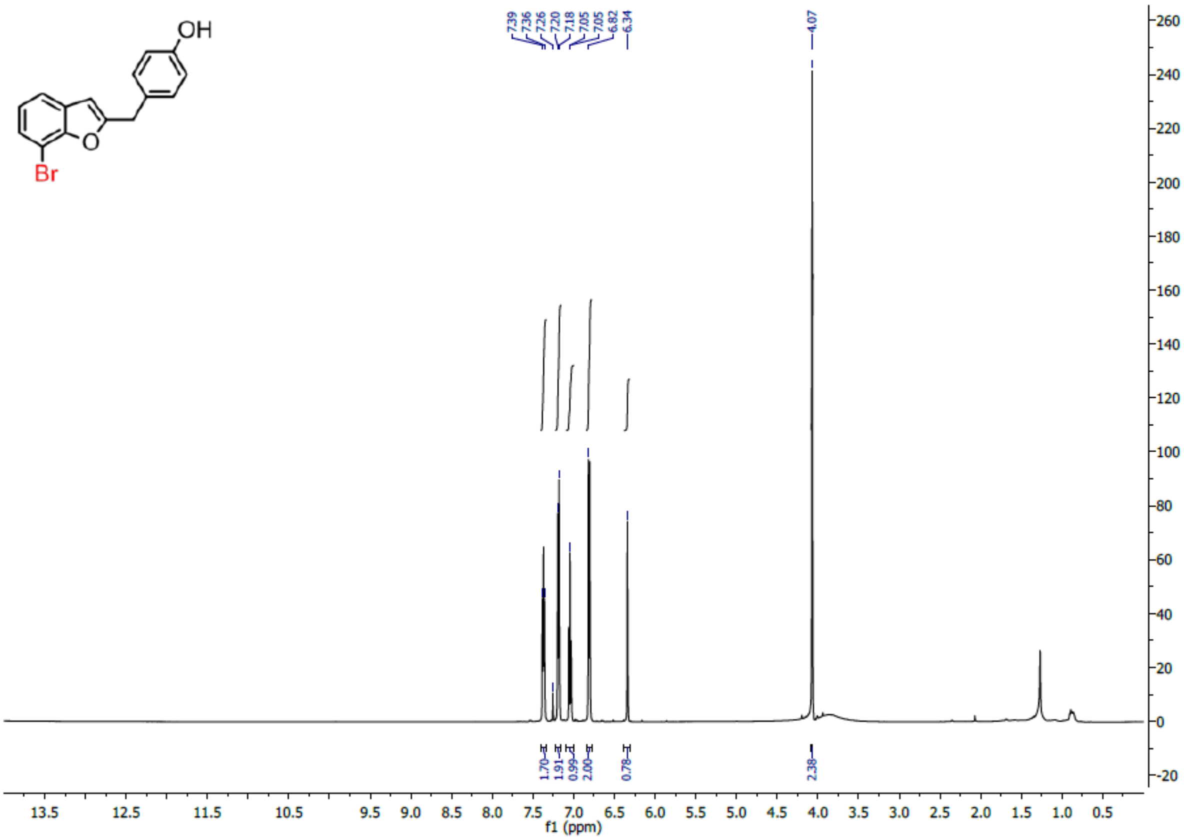

[13] CNMR spectra of *5-Bromo-2-(4-hydroxybenzyl)benzofuran* ***10B***

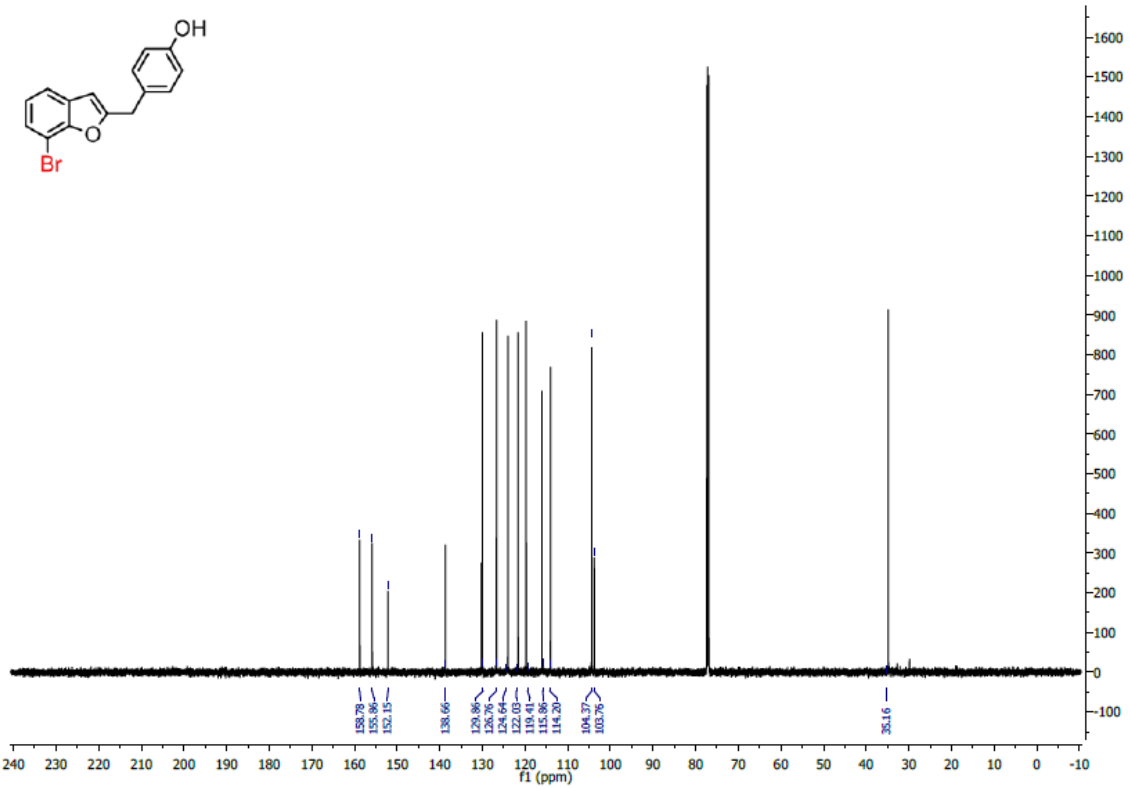

^{1}H NMR spectra of *5-Bromo-2-(4-hydroxybenzyl)benzofuran* ***11B***

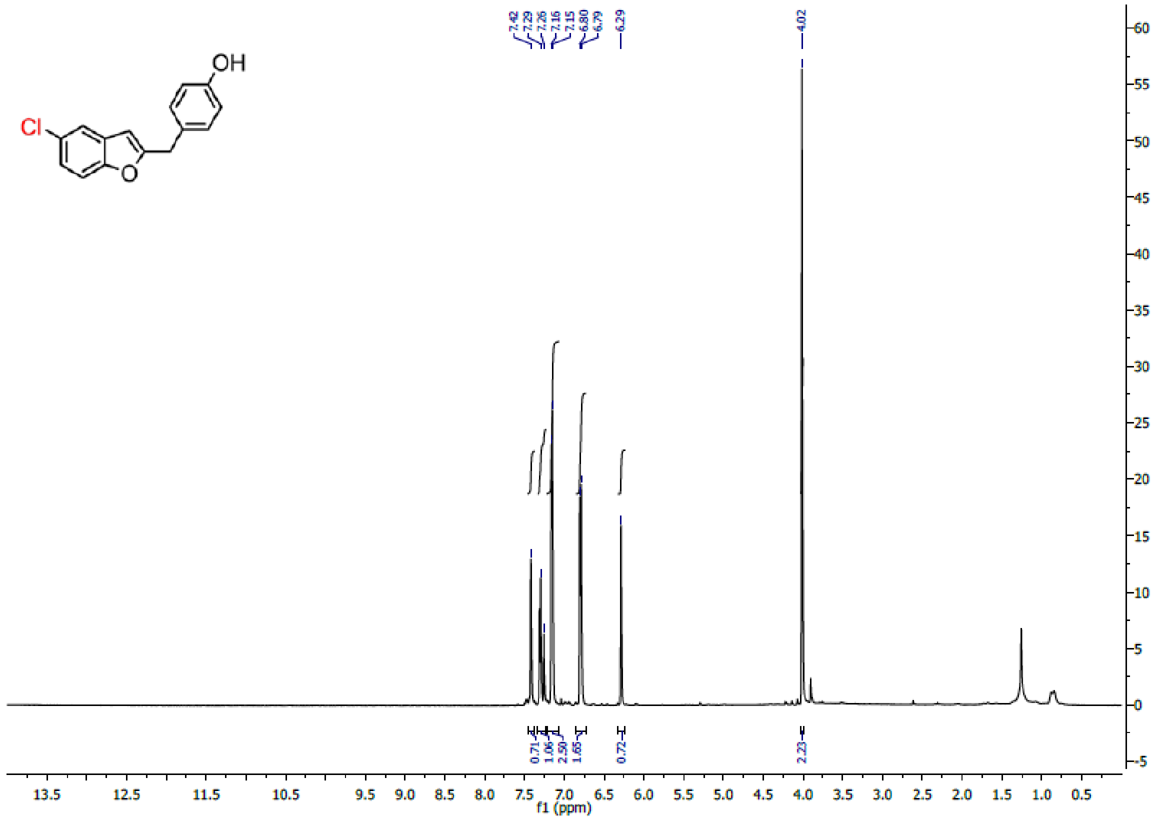

[13] CNMR spectra of *5-Bromo-2-(4-hydroxybenzyl)benzofuran* ***11B***

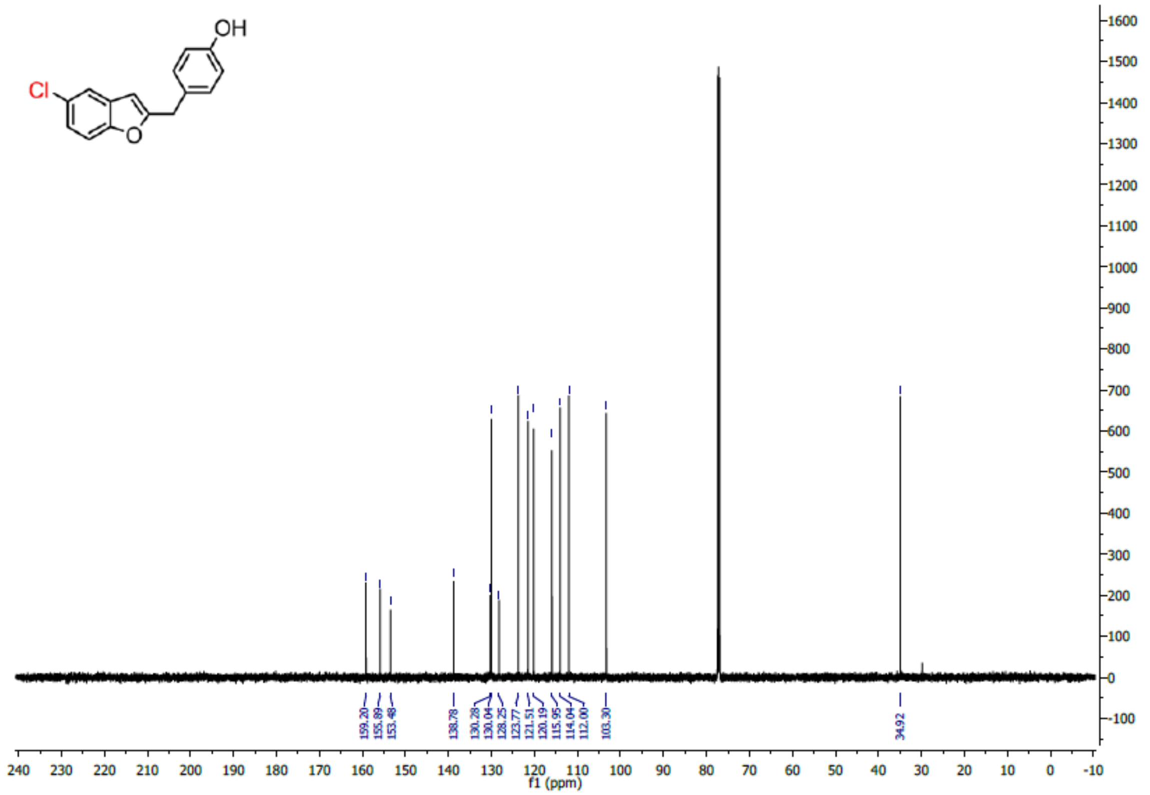

^{1}H NMR spectra of *5-Bromo-2-(4-hydroxybenzyl)benzofuran* ***12B***

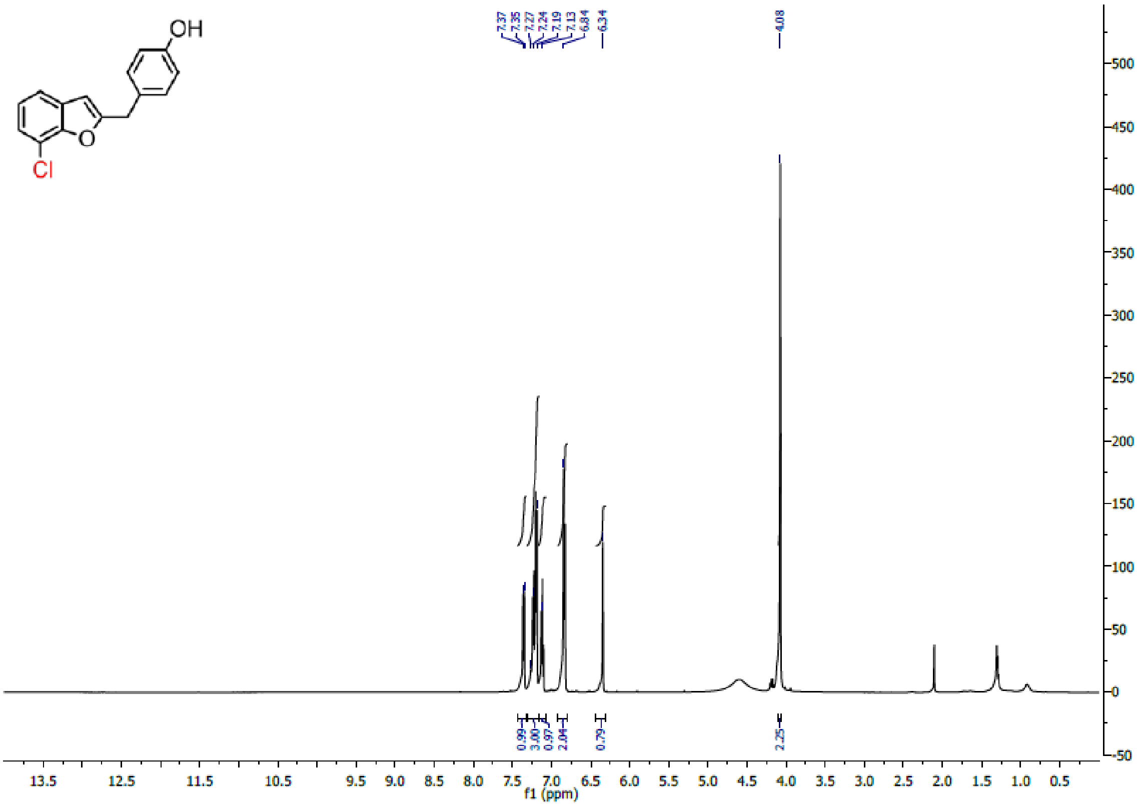

[13] CNMR spectra of *5-Bromo-2-(4-hydroxybenzyl)benzofuran* ***12B***

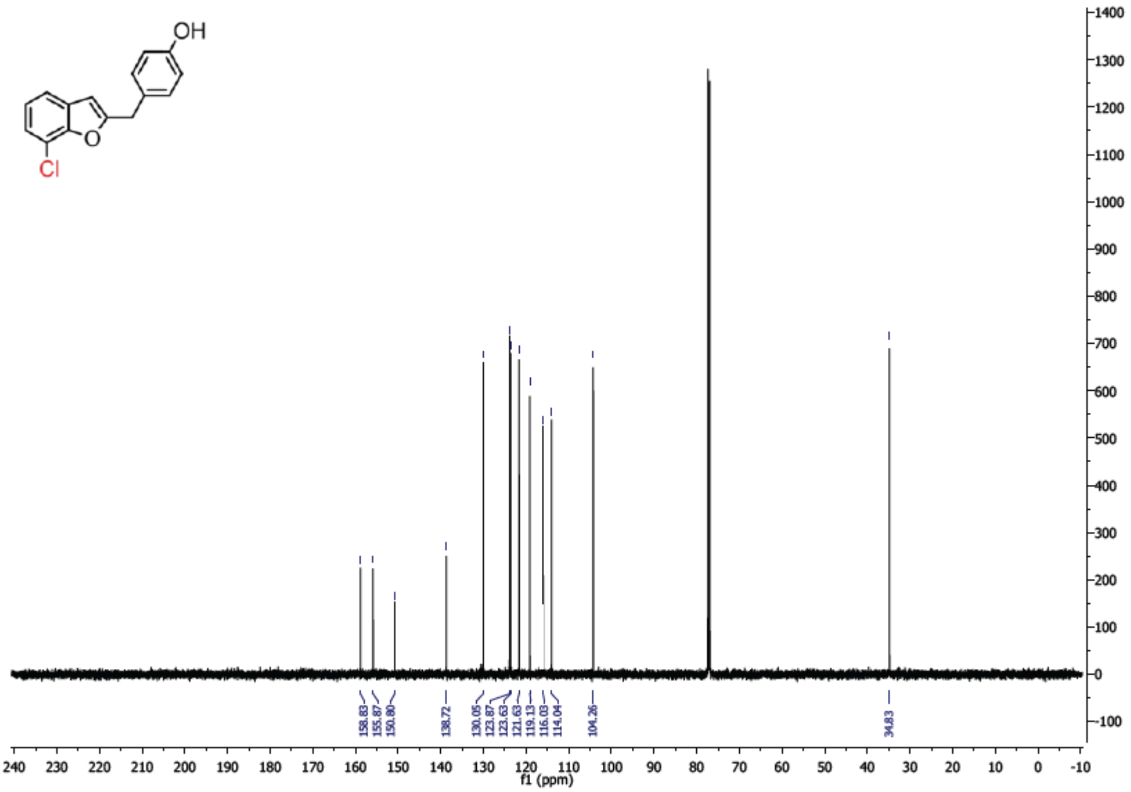

^{1}H NMR spectra of *5-Bromo-2-(4-hydroxybenzyl)benzofuran* ***13B***

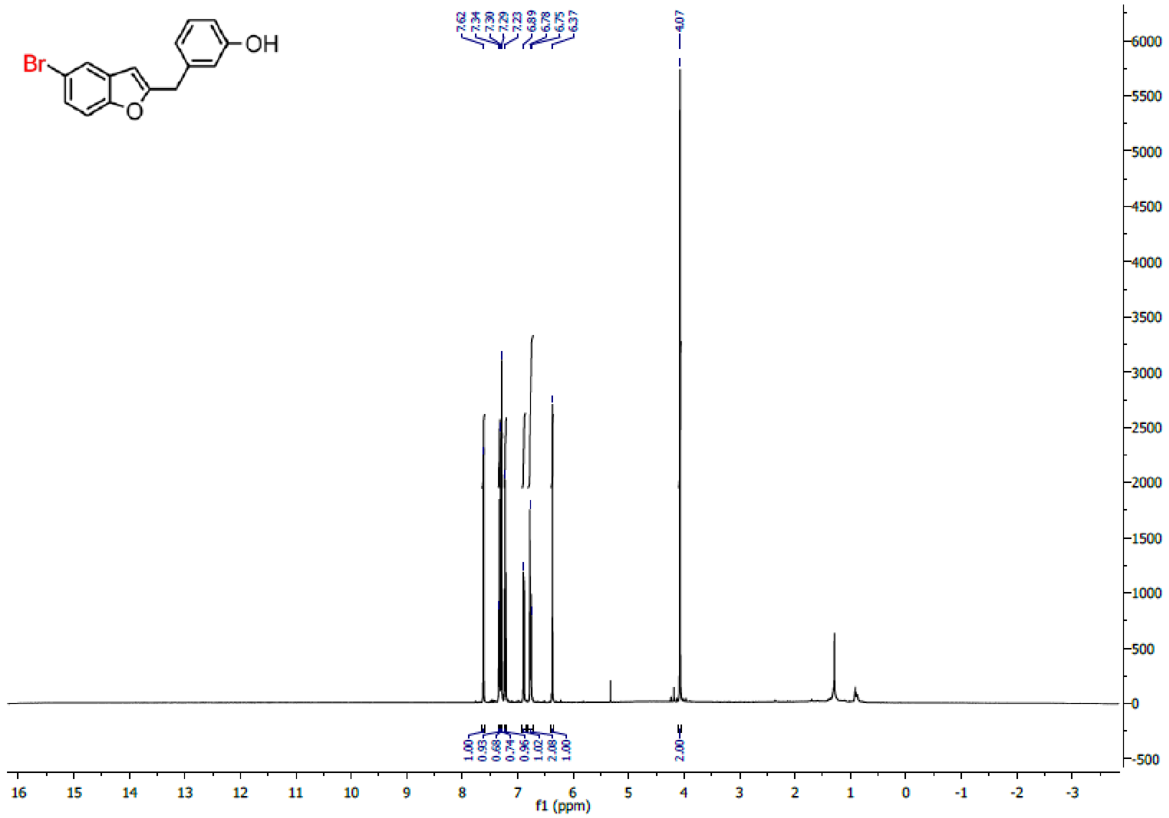

^{13} CNMR spectra of *5-Bromo-2-(4-hydroxybenzyl)benzofuran* ***13B***

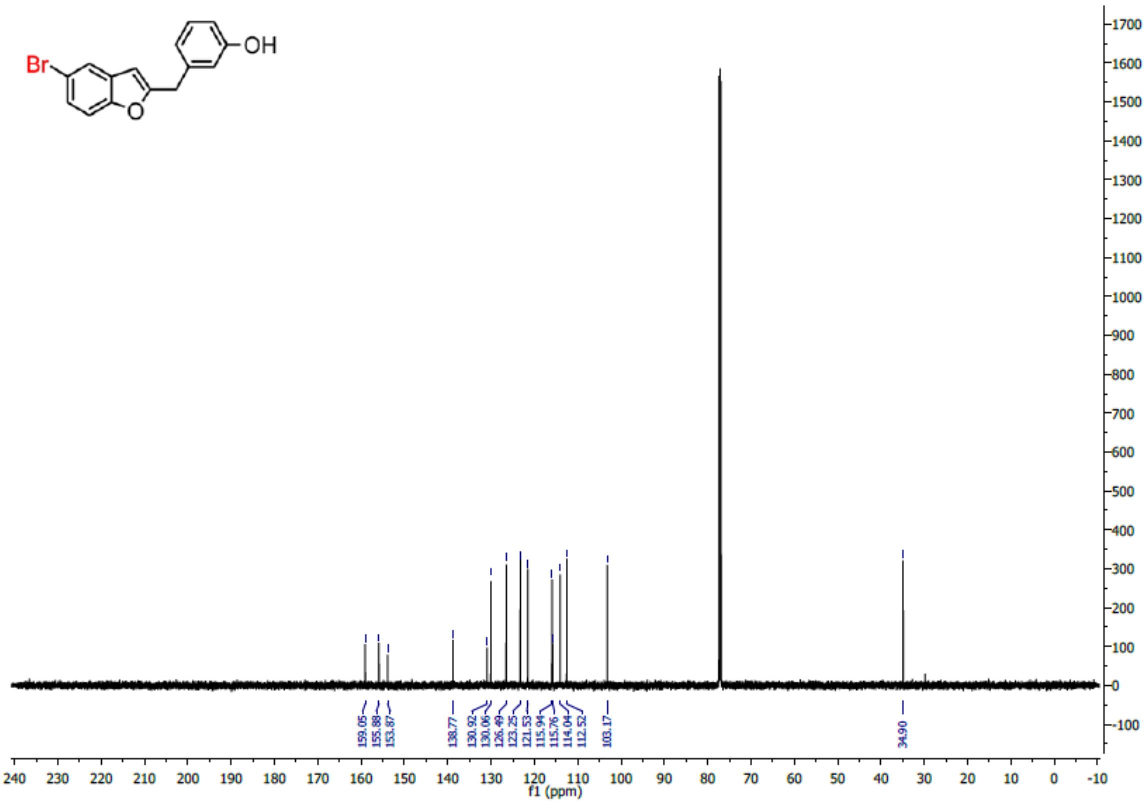

^{1}H NMR spectra of *5-Bromo-2-(4-hydroxybenzyl)benzofuran* ***14B***

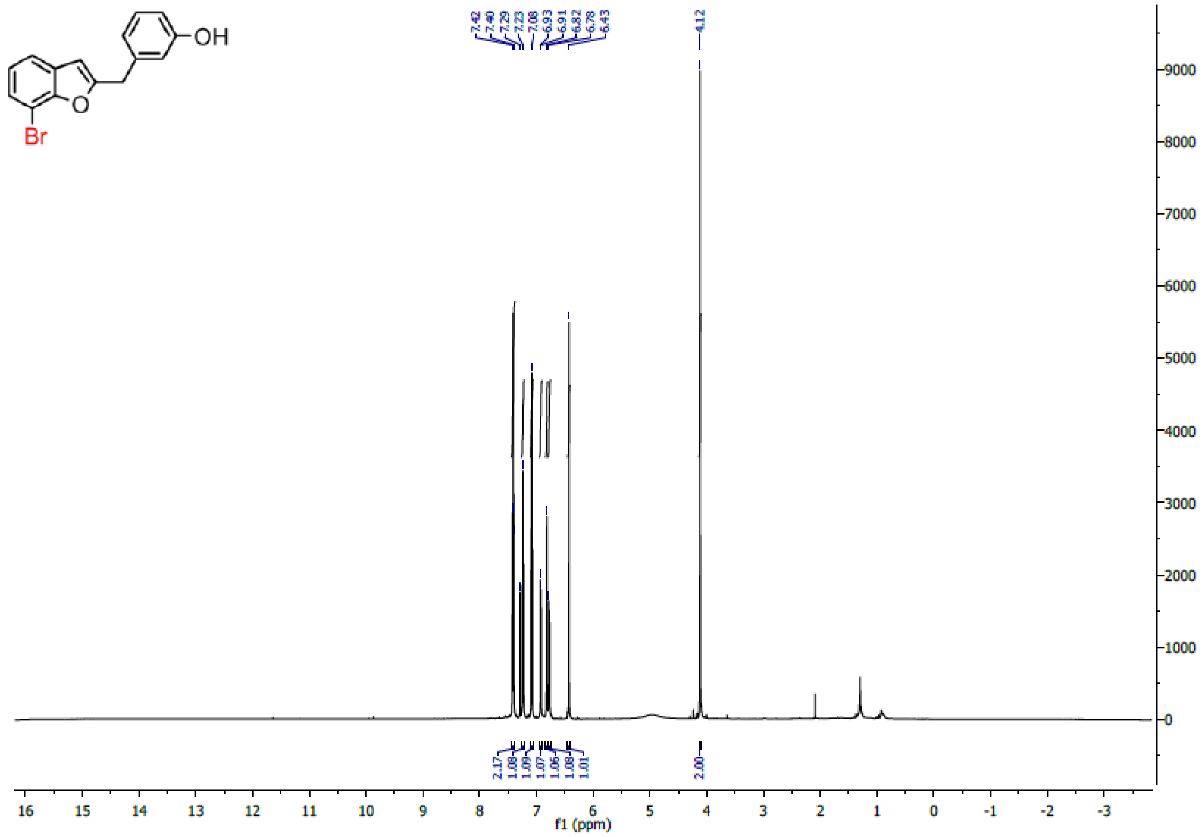

[13] CNMR spectra of *5-Bromo-2-(4-hydroxybenzyl)benzofuran* ***14B***

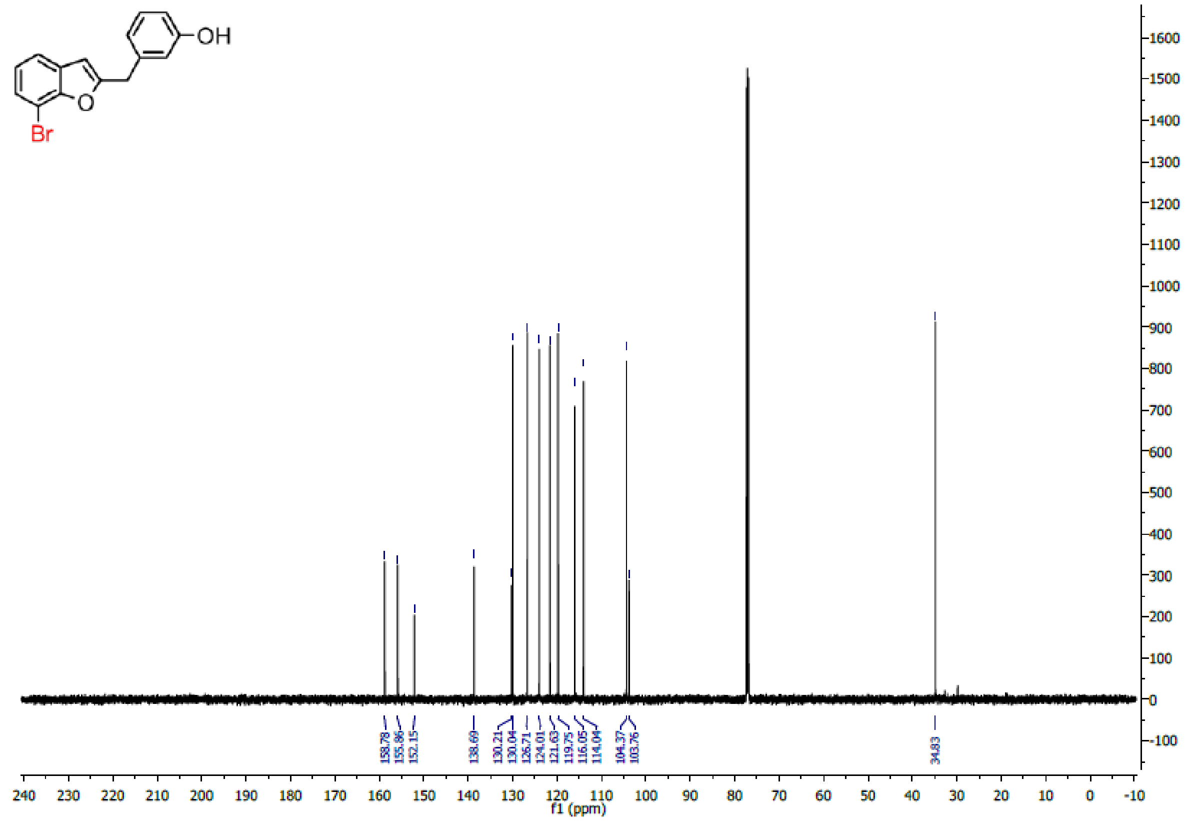

^{1}H NMR spectra of *5-Bromo-2-(4-hydroxybenzyl)benzofuran* ***15B***

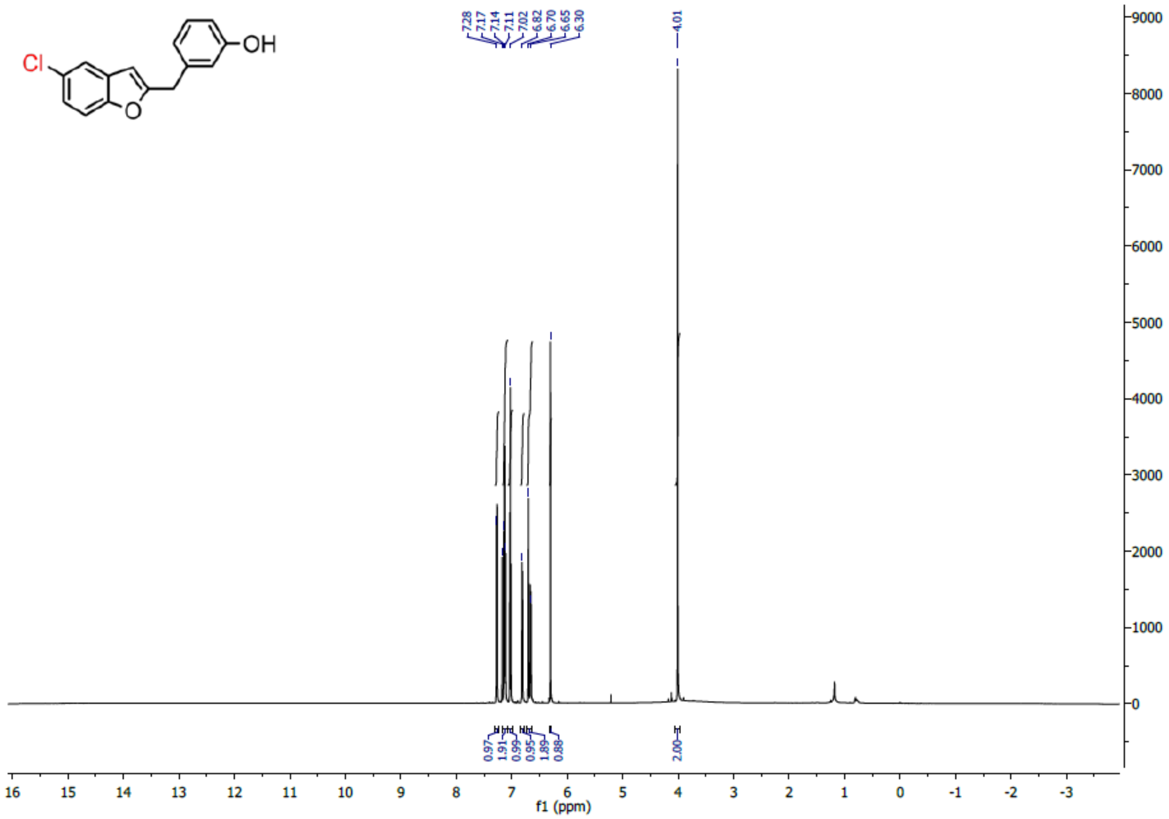

[13] CNMR spectra of *5-Bromo-2-(4-hydroxybenzyl)benzofuran* ***15B***

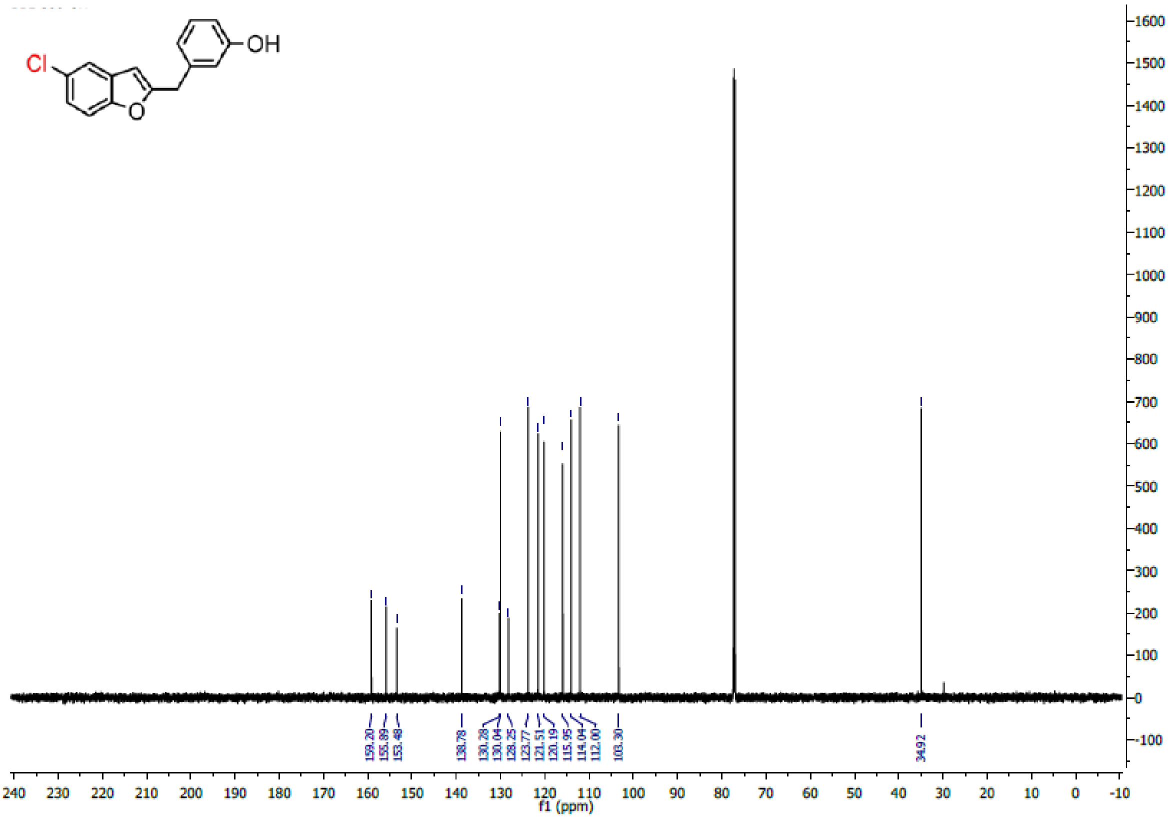

^{1}H NMR spectra of *5-Bromo-2-(4-hydroxybenzyl)benzofuran* ***16B***

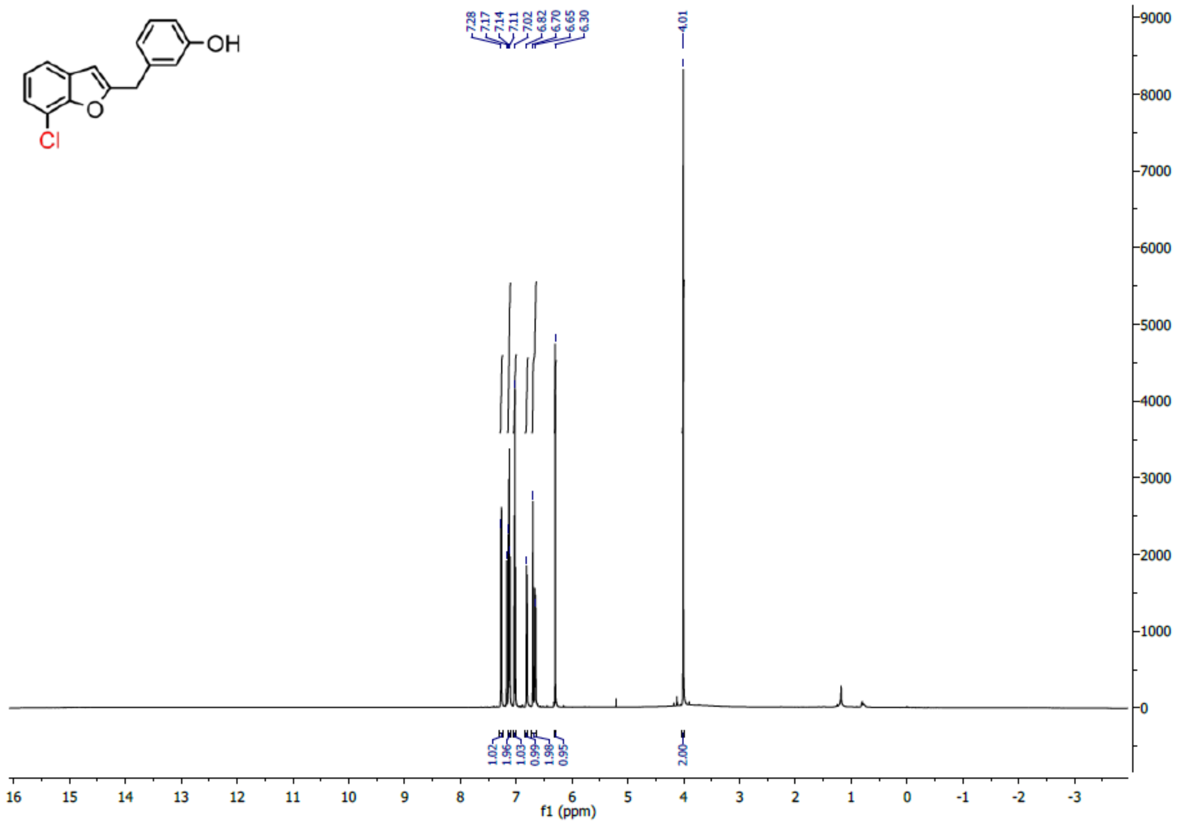

[13] CNMR spectra of *5-Bromo-2-(4-hydroxybenzyl)benzofuran* ***16B***

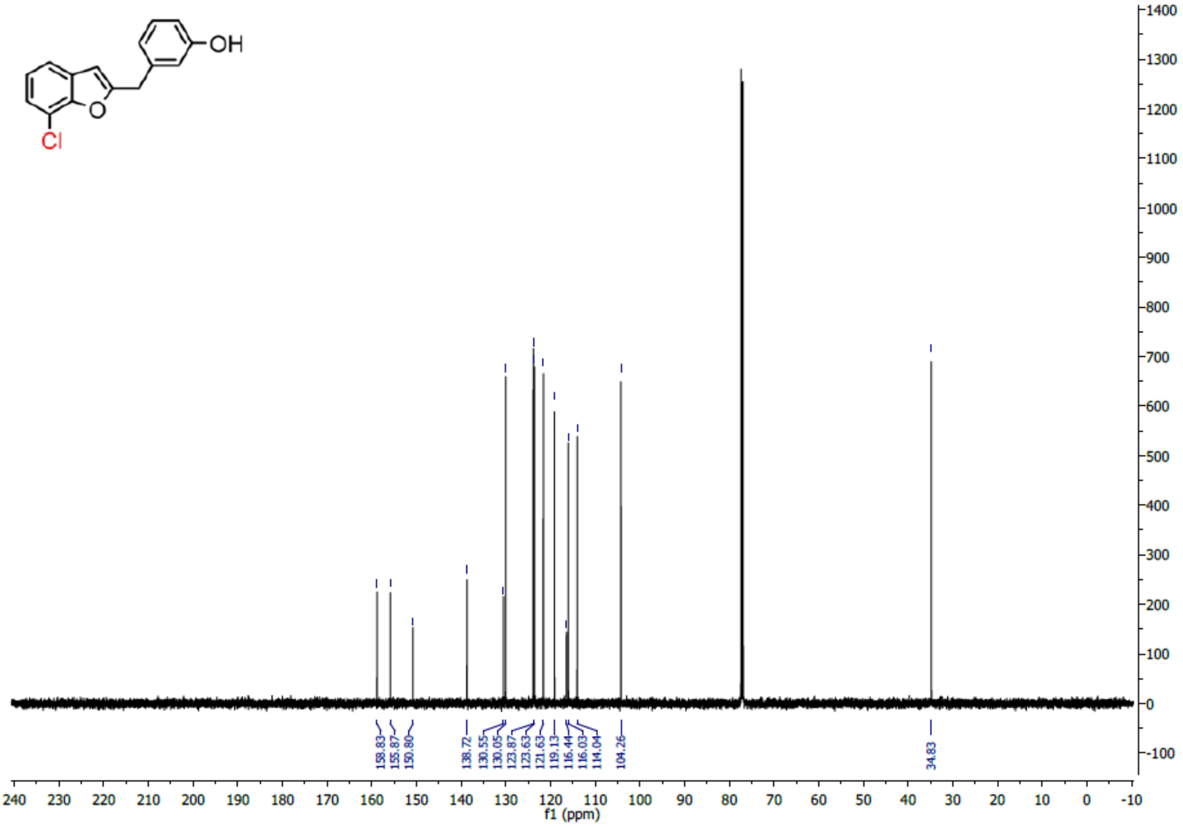

2. Molecular modelling studies

Table S.2.1. Effect of spacer on the docking and IC_{50} values of compounds Table S1

	**A** (no spacer)		**B** (with spacer)	
Ligands	ΔG (kcal/mol)	IC_{50} (μM)	ΔG (kcal/mol)	IC_{50} (μM)
Compound 9	-7.02	>100	-8.81	2.93
Compound 10	-7.52	82.5	-7.80	32.6
Compound 11	-7.58	>100	-7.64	55.66
Compound 12	-7.53	30.3	-7.70	42.98
Compound 13	-7.54	10.6	-7.70	39.95
Compound 14	-7.68	7.96	-7.93	31.37
Compound 15	-7.46	10.52	-7.46	27.84
Compound 16	-7.53	13.42	-8.31	61.65

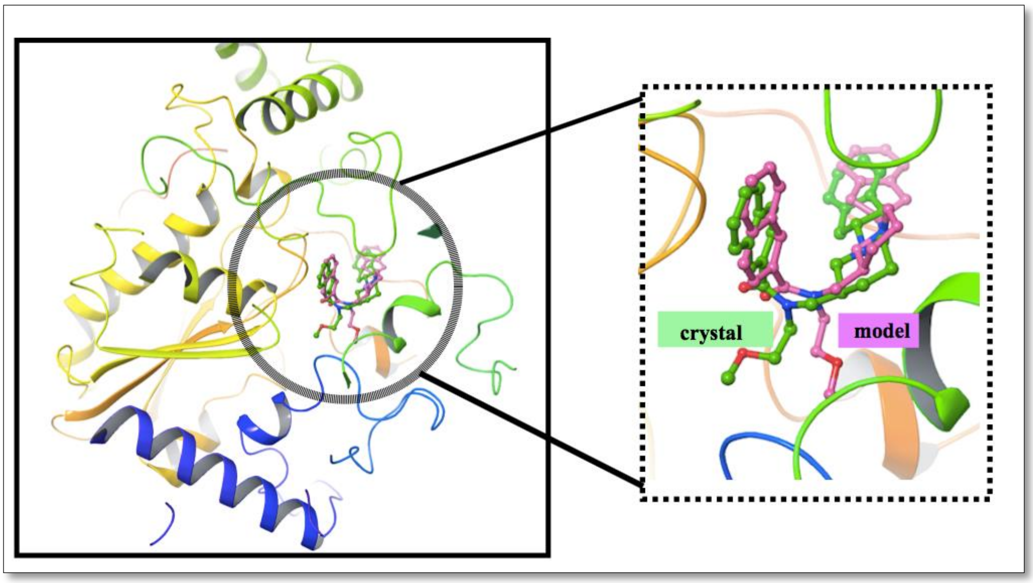

Figure S.2.1. Superimposition of the reference complex (PDB code: 4TPK) and the best docked ligand pose obtained from Swissdock webserver. Figure S1
